# Fluorescent Peptide Tracers for Simultaneous Oxytocin Receptor Activation and Visualization

**DOI:** 10.1002/anie.202515180

**Published:** 2025-09-26

**Authors:** Monika Perisic Böhm, Predrag Kalaba, Rachel S. Gormal, Maja Zupančič, Alexandra Wolf, Mia Juračić, Thomas Kremsmayr, Frédéric A. Meunier, Thierry Langer, Christian W. Gruber, Erik Keimpema, Markus Muttenthaler

**Affiliations:** ^1^ Institute of Biological Chemistry Faculty of Chemistry University of Vienna Vienna Austria; ^2^ Vienna Doctoral School in Chemistry University of Vienna Vienna Austria; ^3^ Clem Jones Centre for Ageing Dementia Research Queensland Brain Institute The University of Queensland Brisbane Australia; ^4^ Department of Molecular Neurosciences Center for Brain Research Medical University of Vienna Vienna Austria; ^5^ School of Biomedical Sciences The University of Queensland Brisbane Australia; ^6^ Department of Pharmaceutical Sciences Faculty of Life Sciences University of Vienna Vienna Austria; ^7^ Institute of Pharmacology Center for Physiology and Pharmacology Medical University of Vienna Vienna Austria; ^8^ Institute for Molecular Bioscience The University of Queensland Brisbane Australia

**Keywords:** Bioactive peptide tracers, Imaging, Immunocytochemistry, Oxytocin receptor visualization, Structure–activity relationship (SAR) study

## Abstract

The oxytocin receptor (OTR) regulates critical physiological functions and has been implicated in a range of diseases, including psychiatric and neurodevelopmental disorders such as autism spectrum disorder. However, a lack of reliable molecular tools hampers the progress in understanding OTR's mechanistic roles in (patho)physiological processes. In this work, we addressed this gap and developed potent, selective, and bright fluorescent peptide tracers that enable precise spatial and functional investigations of OTR actions. Our tracers showed efficient OTR labeling, activation, and internalization in cellular bioassays in both live and fixed overexpression and primary cell systems, including those subjected to immunocytochemical protocols, highlighting their versatility as reliable new imaging tools. Additionally, they facilitated single‐molecule tracking of OTR with live‐cell super‐resolution microscopy and were able to separate OTR‐positive cells from mixed oxytocin and vasopressin receptor‐containing cell populations via fluorescence‐activated cell sorting, underscoring their wider scope for live‐cell applications. In summary, we developed versatile fluorescent tracers based on the endogenous ligand oxytocin for both live‐cell and post‐hoc imaging that have additional functional capabilities beyond traditional antibody labeling, offering new avenues to explore OTR's role in health and disease.

## Graphical abstract

**Illuminating the Oxytocin Receptor**: Here we present the development of fluorescent peptide tracers for the simultaneous visualization and activation of the oxytocin receptor, an important G protein‐coupled receptor involved in health and disease. These tracers are powerful new tools to support various imaging and functional studies across biological systems.

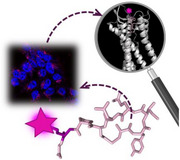

## Introduction

G protein‐coupled receptors (GPCRs) constitute the largest family of membrane receptors and are a key focus of drug development.^[^ ^1^ ^]^ Approximately 35% of approved drugs target GPCRs, highlighting the druggability and therapeutic relevance of this receptor class.^[^ ^2^ ^]^ The oxytocin receptor (OTR) is a prototypical GPCR that is activated by its endogenous peptide ligand oxytocin (OT).^[^ ^3^, ^4^, ^5^, ^6^, ^7^ ^]^ OTR is well‐known for regulating reproductive processes and complex social behaviors and plays an important role in stress responses, cardiovascular function, inflammation, and wound healing.^[^ ^4^, ^5^, ^6^, ^7^ ^]^ This variety of physiological functions renders OTR a compelling target for clinical research and therapeutic interventions.^[^ ^6^, ^7^, ^8^, ^9^, ^10^, ^11^ ^]^ Despite its involvement in cancer,^[^ ^10^, ^12^ ^]^ pain,^[^ ^9^, ^13^ ^]^ and psychiatric,^[^ ^7^ ^]^ and neurodevelopmental disorders,^[^ ^14^ ^]^ current clinical applications targeting OTR are largely confined to the reproductive field.^[^ ^4^, ^15^, ^16^, ^17^ ^]^ Although considerable efforts have been made by both industry and academia, the precise sites of OTR action and its molecular mechanisms in many physiological functions remain poorly understood. This is primarily due to a lack of well‐validated molecular tools to specifically study OTR location and signaling to advance our understanding of its role in (patho)physiological processes and facilitate the development of OTR‐specific therapeutics.

**Figure 1:**
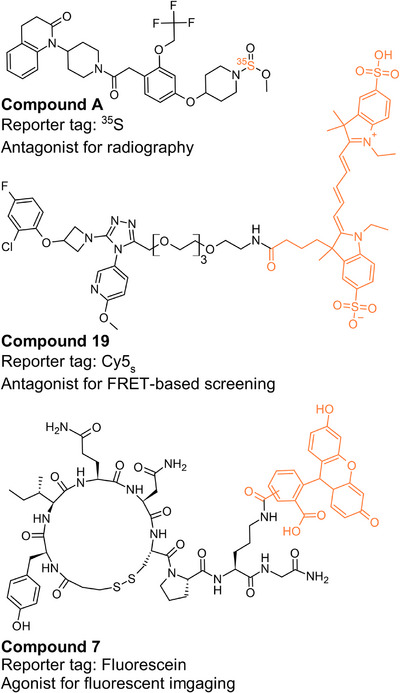
Overview of the most selective OTR tracers. Compound A,^[^ ^19^ ^]^ compound 19,^[^ ^25^ ^]^ and compound 7.^[^ ^27^ ^]^

The high homology between OTR and its closely related vasopressin receptors (VPRs; V_1a_R, V_1b_R, V_2_R),^[^ ^18^ ^]^ along with the structural similarities between the two endogenous ligands OT (CY**I**QNCP**L**G) and vasopressin (VP; CY**F**QNCP**R**G), present significant challenges in developing selective ligands^[^ ^10^, ^15^ ^]^ and antibodies.^[^ ^3^, ^10^ ^]^ Despite decades of research, no knockout‐verified OTR‐selective antibodies are commercially available. While OTR‐selective radioligands such as compound A, a ^35^S‐labeled OTR‐selective small‐molecule antagonist (Figure 1),^[^ ^19^ ^]^ have been developed, their spatial resolution with radiography is limited.^[^ ^3^ ^]^

Fluorescently labeled tracers represent an alternative class of reporter tools^[^ ^20^, ^21^ ^]^ that, when combined with state‐of‐the‐art microscopy, support high‐resolution mapping and quantification of protein/receptor expression sites,^[^ ^22^ ^]^ trafficking studies,^[^ ^23^ ^]^ and interaction studies (e.g., via fluorescence and bioluminescence resonance energy transfer approaches, FRET and BRET).^[^ ^24^ ^]^ In addition, their application as pharmacological tools in time‐resolved FRET studies can support high‐throughput screening platforms.^[^ ^25^ ^]^ However, the few reported fluorescent tracers for OTR exhibit limitations, including poor OTR selectivity, suboptimal fluorophore properties (e.g., photolabile, pH‐sensitive), and non‐fixable designs^[^ ^15^, ^21^, ^26^, ^27^ ^]^ (Figure 1), highlighting the need for more advanced tools.

In this work, we used an OT‐like peptide as the basis for tracer development and progressively improved OTR selectivity by modifying the linker between the peptide and bright, photostable fluorophores. This linker‐centric design strategy yielded selective tracers capable of both visualizing and activating OTR, offering a functional advantage over classical antibody‐based tools, which are limited to visualization. These tracers support a wide range of sophisticated and broadly applicable techniques, including high‐ and super‐resolution imaging, single‐molecule tracking in live cells, fluorescence‐activated cell sorting (FACS), and post‐fixation immunocytochemical analysis. These new versatile tools enable detailed investigation of OTR biology in both live and fixed systems and open new avenues for studying its roles in health and disease.

## Results

### OTR Tracer Design

**Figure 2:**
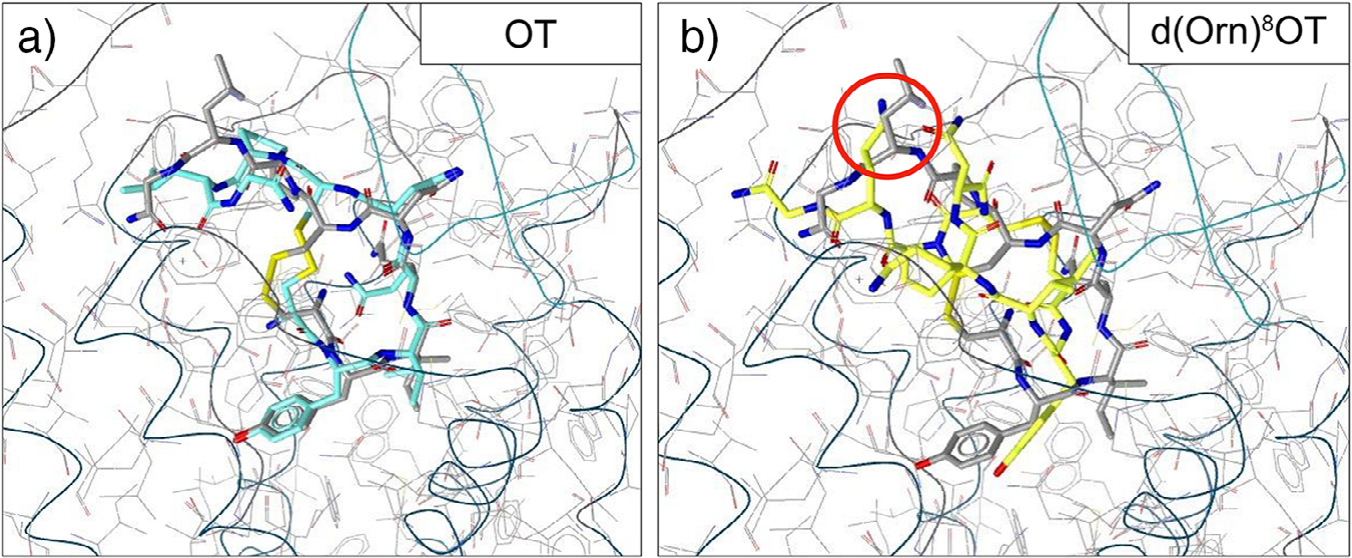
Docking of OT and d(Orn)^8^OT to the human OTR binding pocket. a) OT‐hOTR (7RYC; *grey*) overlaid with the lowest energy docked OT structure (*cyan*). The amino acids of both macrocycles align well, with only Leu^8^ pointing in a different direction. Comparable geometries and highly similar LigandScout binding affinity score values of −30.86 and −30.76 for the cryo‐EM and docked OT, respectively, led to the conclusion of a valid docking approach. b) OT‐hOTR (*grey*), overlaid with a low‐energy docked d(Orn)^8^OT structure (*yellow*). The Orn^8^ side chain was pointing from the binding pocket outwards, supporting the hypothesis that this position (*red circle*) could be used for linker attachment of other moieties without interfering with the active site. LigandScout binding affinity score of −30.60 indicated only a slightly weaker binding affinity to hOTR.

We used endogenous OT as the starting point based on the following considerations: 1) We envisioned that a bioactive tracer would not only be able to visualize OTR but also provide valuable information on signaling, internalization, and trafficking, thereby providing an edge over antagonists and antibodies. 2) Peptides have sufficient surface area and chirality to achieve OTR selectivity, while small‐molecule agonists often lack selectivity against VPRs (selectivity defined as >100‐fold difference in K_i_).^[^ ^16^, ^17^, ^28^ ^]^ 3) The recent cryo‐EM structure of the OT‐OTR complex provides detailed molecular insights facilitating tracer design.^[^ ^29^, ^30^ ^]^ Considering the OT‐OTR structure and structure‐activity relationship (SAR) data,^[^ ^17^, ^31^, ^32^, ^33^, ^34^, ^35^, ^36^ ^]^ we chose Leu^8^ of OT as our preferred modification site. Leu^8^ points towards the extracellular space from the OTR binding pocket, providing enough space for a linker and fluorophore without compromising affinity or activity.^[^ ^12^, ^21^, ^30^ ^]^ Orn^8^ or Lys^8^ modifications have been used successfully in the past to introduce labeling groups,^[^ ^21^ ^]^ and the attachment of fluorescein to Orn^8^ improved OTR selectivity.^[^ ^27^ ^]^ Deamination of the N‐terminus of OT (dOT) results in enhanced metabolic stability and a more hydrophobic N‐terminus, leading to a better binding fit.^[^ ^35^ ^]^ Therefore, d(Orn)^8^OT was investigated for its suitability as a parent structure. To confirm a favorable position within the OTR binding pocket, we performed docking studies with OT and d(Orn)^8^OT using AutoDock Vina 1.1^[^ ^37^ ^]^ implemented in LigandScout^[^ ^38^ ^]^ (Figure 2). After validating our approach by comparison of OT docked to OTR with the OT‐OTR cryo‐EM complex (Figure 2a),^[^ ^30^ ^]^ we docked d(Orn)^8^OT. According to the obtained low‐energy binding position, d(Orn)^8^OT only had slightly decreased binding affinity compared to OT and the Orn^8^ side chain pointed towards the extracellular space (Figure 2b), supporting a suitable attachment point for linkers and fluorophores and d(Orn)^8^OT as a good starting point for our tracer design.

### Linker Design and Fluorophore Choice

**Figure 3:**
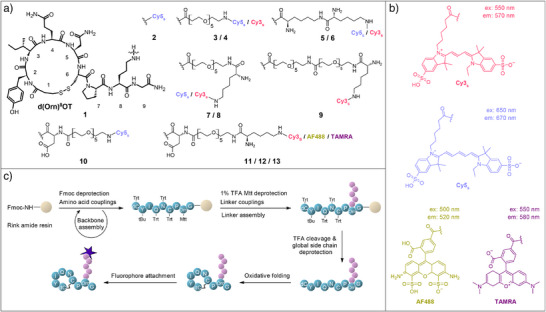
Molecular structure of d(Orn)^8^OT parent peptide and fluorophores and fluorescent tracer synthesis. a) Overview of synthesized tracer structures. The d in d(Orn)^8^OT stands for desamino (N‐terminal α‐amino group absent). Note: The chemical structure is shown with cis amide bonds for illustration purposes only and does not reflect the actual amide bond configuration, which is trans. b) Chemical structures of the Cy3_s_, Cy5_s_, AF488, and TAMRA fluorophores. c) Synthetic strategy to produce the different fluorescent OTR tracers. Backbone and linker assemblies were carried out via Fmoc‐SPPS, followed by TFA global side chain deprotection and resin cleavage, oxidative folding, and in‐solution fluorophore attachment using NHS‐activated dyes.

Given that all amino acids of the parent peptide are involved in receptor binding^[^ ^30^ ^]^ and the sensitivity to minor structural changes,^[^ ^35^ ^]^ we focused on the linker design as a new strategy for obtaining potent and selective tracers. Polyethylene glycol (PEG) was chosen as a spacer between the parent peptide and the fluorophore due to its water solubility, flexibility, chemical inertness, metabolic stability, and lack of toxicity or immune response. Based on our alignment of d(Orn)^8^OT‐[PEG_5_]^8^ with OTR using PyMOL (Figure S1), PEG_5_ was estimated to be sufficiently long for the fluorophore to protrude from the binding pocket, minimizing steric hindrance and receptor interactions that could disrupt binding. We incorporated an enzymatically stable d‐lysine (d‐Lys, k) into the linker design to support immunocytochemistry (ICC) applications, where a free amine or other nucleophile is required to enable the formaldehyde‐based crosslinking step to immobilize tracer and receptor for post‐hoc imaging and analysis.^[^ ^39^ ^]^ d(Orn)^8^OT itself does not contain any available nucleophilic moieties since the two cysteine residues form a disulfide bond, and the N‐terminal amine was removed for a better binding fit. Including d‐Lys solved this problem, allowing the ε‐amine to be used for linker extension or fluorophore attachment and the α‐amine for formaldehyde‐based cross‐linking (Figure 3a).

As fluorophores, we selected sulfonated carbocyanine (Cy_s_) dyes due to their excellent photo‐ and pH‐stability, brightness and water solubility, low tendency to form self‐quenching aggregates, and commercial availability.^[^ ^40^, ^41^ ^]^ More specifically, we chose Cy3_s_ with absorption and emission within the central range of the visible spectrum (ex: 550 nm, em: 570 nm) and Cy5_s_ in the red spectral range (ex: 650 nm, em: 670 nm).^[^ ^41^ ^]^ To further expand the chemical diversity of fluorophores, Alexa Fluor 488 (AF488, ex: 500 nm, em: 520 nm) and TAMRA (ex: 550 nm, em: 580 nm) were additionally incorporated into the series (Figure 3b).^[^ ^42^, ^43^ ^]^ Since all these fluorophores are well‐established and extensively characterized in the literature, no additional assessment of their physicochemical properties was performed in this study.

### OTR Tracer Synthesis

We assembled the peptide‐linker precursors through manual Fmoc‐SPPS (9‐fluorenylmethyloxycarbonyl solid‐phase peptide synthesis) followed by trifluoroacetic acid (TFA) cleavage, oxidative folding, purification, and in‐solution fluorophore attachment (Figures 3c and S2). Fluorophores were attached as NHS esters to the peptide‐linker constructs via amide bond formation. The fluorophore‐labeled tracers were characterized by high‐resolution electrospray ionization mass spectrometry (HR‐ESI‐MS) and analytical reversed‐phase high‐performance liquid chromatography (RP‐HPLC; Supporting Information). All synthesized compounds had a purity of >95%.

### Structure‐Affinity Relationship Study

The binding affinity and selectivity towards OTR versus VPRs (V_1a_R, V_1b_R, V_2_R) were determined through radioligand displacement assays (Table 1, Figure S3). Due to the stepwise attachment of the different linker components in tracers **2**–**13**, changes in binding affinities could be assigned to the newly introduced moieties, delivering insightful structure‐affinity relationship data. The parent peptide and all tracers retained excellent OTR binding affinity in the low nanomolar range or below (K_i_: 0.4–22.6 nM); however, the affinity to the three VPRs varied substantially. The parent peptide d(Orn)^8^OT (**1**) displayed no OTR preference, interacting with OTR, V_1a_R, and V_1b_R to equal extents. Only the binding affinity towards V_2_R was significantly weaker, a trend that remained unchanged throughout tracer development. Direct attachment of Cy5_s_, yielding d(Orn)^8^OT‐[Cy5_s_]^8^ (**2**), enhanced preference for OTR over V_1a_R (26‐fold) and V_1b_R (96‐fold). Introducing PEG_5_ between the peptide and fluorophore further improved OTR selectivity, yielding d(Orn)^8^OT‐[PEG_5_‐Cy5_s_]^8^ (**3**) and d(Orn)^8^OT‐[PEG_5_‐Cy3_s_]^8^ (**4**) with ∼50‐fold over V_1a_R and >200‐fold over V_1b_R. Introducing metabolically stable d‐Lys for cross‐linking applications, yielding d(Orn)^8^OT‐[k^ε^‐k^ε^‐Cy5_s_]^8^ (**5**) and d(Orn)^8^OT‐[k^ε^‐k^ε^‐Cy3_s_]^8^ (**6**), enhanced binding to V_1a_R and V_1b_R, thereby reducing OTR selectivity. Comparing the selectivity difference between the *neutral* PEG linker and the *positively charged* d‐Lys linker (at physiological pH) suggested that a positive charge supported V_1a_R and V_1b_R binding, as also proposed by others.^[^ ^21^, ^26^ ^]^ This also aligned with the positively charged Arg^8^ residue in VP compared to neutral Leu^8^ in OT and the lack of selectivity of d(Orn)^8^OT (again with a positive charge at position 8). In an attempt to rescue OTR preference, we moved the positive charge away from the binding pocket by inserting firstly one and then two PEG_5_ linkers between the parent peptide and d‐Lys, yielding d(Orn)^8^OT‐[PEG_5_‐k^ε^‐Cy5_s_]^8^ (**7**), d(Orn)^8^OT‐[PEG_5_‐k^ε^‐Cy3_s_]^8^ (**8**), and d(Orn)^8^OT‐[PEG_5_‐PEG_5_‐k^ε^‐Cy3_s_]^8^ (**9**). Although OTR selectivity against V_1b_R was regained in all three tracers (>100‐fold), OTR selectivity against V_1a_R was not achieved (9–15‐fold). No differences in binding affinities were observed between attaching either Cy5_s_ or Cy3_s_, indicating that the structural differences between these dyes were too small to result in significant pharmacological changes.

**Table 1:** Binding affinity (K_i_) values of synthesized OTR tracers and reference compounds obtained from radioligand displacement assays using membrane preparations of HEK293 cells stably expressing human (h) or transiently expressing murine (m) OTR, V_1a_R, V_1b_R, or V_2_R and [^3^H]‐OT or [^3^H]‐VP. Average K_i_ values ± SD were calculated from dose‐response curves measured in duplicates in at least three independent experiments. K_i_ ratios express the fold binding selectivity for OTR and were obtained by dividing respective VPR affinities by the OTR affinity. K_i_ values of >10 µM were assigned to compounds that displaced <25% of the radioligand at 10 µM. d = d‐aspartic acid; k = d‐lysine; PEG_5_ = (polyethylene glycol)_5_ chain. k^ε^ = attachment via the epsilon amine. The d in d(Orn)^8^OT stands for desamino (N‐terminal α‐amino group absent).

		K_i_ (nM)				Selectivity ratio		
Compound ID		hOTR	hV_1a_R	hV_1b_R	hV_2_R	$\frac{hV_{1a}R}{hOTR}$	$\frac{hV_{1b}R}{hOTR}$	$\frac{hV_{2}R}{hOTR}$
	Oxytocin (OT)	0.5 ± 0.2	18.3 ± 4.9	588 ± 19	2842 ± 665	37	>1000	>5000
	Vasopressin (VP)	7.9 ± 5.0	0.2 ± 0.1	0.3 ± 0.1	3.6 ± 1.3	0.03	0.04	0.5
	OTR antagonist (OTA)	44.9 ± 8.8	45.8 ± 11.9	>10 000	>10 000	1.02	223	223
	Scrambled tracer (SCR)	>10 000	>10 000	>10 000	>10 000	–	–	–
1	d(Orn)^8^OT	0.7 ± 0.3	0.4 ± 0.1	0.8 ± 0.1	328 ± 23	0.6	1.1	469
2	d(Orn)^8^OT‐[Cy5_s_]^8^	0.9 ± 0.3	23 ± 8	86 ± 20	3,683 ± 13	26	96	>2500
3	d(Orn)^8^OT‐[PEG_5_‐Cy5_s_]^8^	1.3 ± 0.5	70 ± 42	376 ± 108	>10 000	54	289	>5000
4	d(Orn)^8^OT‐[PEG_5_‐Cy3_s_]^8^	1.5 ± 0.6	80 ± 18	372 ± 35	>10 000	53	248	>5000
5	d(Orn)^8^OT‐[k^ε^‐k^ε^‐Cy5_s_]^8^	0.4 ± 0.2	3.8 ± 0.4	13 ± 3	2036 ± 454	10	33	>5000
6	d(Orn)^8^OT‐[k^ε^‐k^ε^‐Cy3_s_]^8^	0.8 ± 0.6	3.3 ± 1.1	10 ± 4	2135 ± 633	4	13	>2500
7	d(Orn)^8^OT‐[PEG_5_‐k^ε^‐Cy5_s_]^8^	1.2 ± 0.6	11 ± 1	176 ± 29	>10 000	9	147	>5000
8	d(Orn)^8^OT‐[PEG_5_‐k^ε^‐Cy3_s_]^8^	1.4 ± 0.6	21 ± 10	242 ± 97	>10 000	15	173	>5000
9	d(Orn)^8^OT‐[PEG_5_‐PEG_5_‐k^ε^‐Cy3_s_]^8^	1.5 ± 0.5	22 ± 1	199 ± 71	>10 000	15	133	>5000
10	d(Orn)^8^OT‐[d‐PEG_5_‐Cy5_s_]^8^	6.0 ± 1.4	553 ± 128	1315 ± 352	>10 000	92	219	>1500
11	d(Orn)^8^OT‐[d‐PEG_5_‐k^ε^‐Cy3_s_]^8^	1.8 ± 0.3	205 ± 107	302 ± 32	>10 000	114	201	>5000
12	d(Orn)^8^OT‐[d‐PEG_5_‐k^ε^‐AF488]^8^	22.6 ± 7.6	1430 ± 381	1800 ± 148	>10 000	63	80	442
13	d(Orn)^8^OT‐[d‐PEG_5_‐k^ε^‐TAMRA]^8^	7.5 ± 1.4	631 ± 143	932 ± 340	>10 000	84	124	>1000

		mOTR	mV_1a_R	mV_1b_R	mV_2_R	$\frac{mV_{1a}R}{mOTR}$	$\frac{mV_{1b}R}{mOTR}$	$\frac{mV_{2}R}{mOTR}$
	Oxytocin (OT)	0.5 ± 0.1	1405 ± 566	416 ± 64	1574 ± 219	>2500	832	>2500
	Vasopressin (VP)	8.1 ± 2.0	1.0 ± 0.4	0.4 ± 0.1	0.5 ± 0.1	0.1	0.05	0.06
	OTR antagonist (OTA)	5.9 ± 1.7	>10 000	>10 000	3009 ± 346	>1500	>1500	510
	Scrambled tracer (SCR)	>10 000	>10 000	>10 000	>10 000	–	–	–
11	d(Orn)^8^OT‐[d‐PEG_5_‐k^ε^‐Cy3_s_]^8^	3.5 ± 0.8	1,311 ± 435	157 ± 11	>10 000	375	45	>2500

Based on the observation that a positive charge close to residue 8 enhances VPR affinity, we decided to introduce a negatively charged d‐aspartic acid (d‐Asp, d) into the linker design to enhance OTR selectivity. The resulting tracer d(Orn)^8^OT‐[d‐PEG_5_‐Cy5_s_]^8^ (**10**) displayed 90‐fold OTR preference over V_1a_R and >200‐fold selectivity over V_1b_R, validating our approach. Combining our most successful approaches finally resulted in d(Orn)^8^OT‐[d‐PEG_5_‐k^ε^‐Cy3_s_]^8^ (**11**) with a 2 nM OTR affinity and >100‐fold OTR selectivity over V_1a_R, >150‐fold selectivity over V_1b_R, and >5000‐fold selectivity over V_2_R. To further investigate whether the observed selectivity was attributable solely to the linker or a combination of linker and dye, the fluorophores AF488 and TAMRA were incorporated. Although d(Orn)^8^OT‐[d‐PEG_5_‐k^ε^‐AF488]^8^ (**12**) and d(Orn)^8^OT‐[d‐PEG_5_‐k^ε^‐TAMRA]^8^ (**13**) exhibited slightly reduced selectivity (63‐ and 84‐fold over V_1a_R, respectively), detailed analysis of their binding affinities revealed a general shift of all four affinities toward the higher nanomolar to micromolar range. These findings indicated that, while the fluorophore did influence the binding properties, the linker remained the primary determinant of selectivity for OTR over VPRs. To identify receptor residues involved in mediating binding selectivity, an additional molecular docking study was performed. However, docking d(Orn)^8^OT‐[PEG_5_‐k^ε^‐Cy3_s_]^8^ (**8**) and d(Orn)^8^OT‐[d‐PEG_5_‐k^ε^‐Cy3_s_]^8^ (**11**) did not yield binding poses in which the macrocycles adopted reasonable orientations within the binding pocket (Figure S5).

Given the well‐documented species differences in ligand affinity and selectivity for OTR and VPRs,^[^ ^4^, ^44^, ^45^ ^]^ we also performed binding assays at the murine receptors (Table 1). While binding affinities of reference compounds OT, VP, and OTA were consistent with literature,^[^ ^44^, ^46^ ^]^ the human OTR‐selective (114‐fold) lead compound (**11**) exhibited reduced selectivity for the murine OTR (45‐fold) due to an increased binding affinity at murine V_1b_R, which should be taken into consideration when applying the tracer to samples from murine origin.

### OTR Selectivity and Biological Activity of Selected Compounds In Vitro

**Figure 4:**
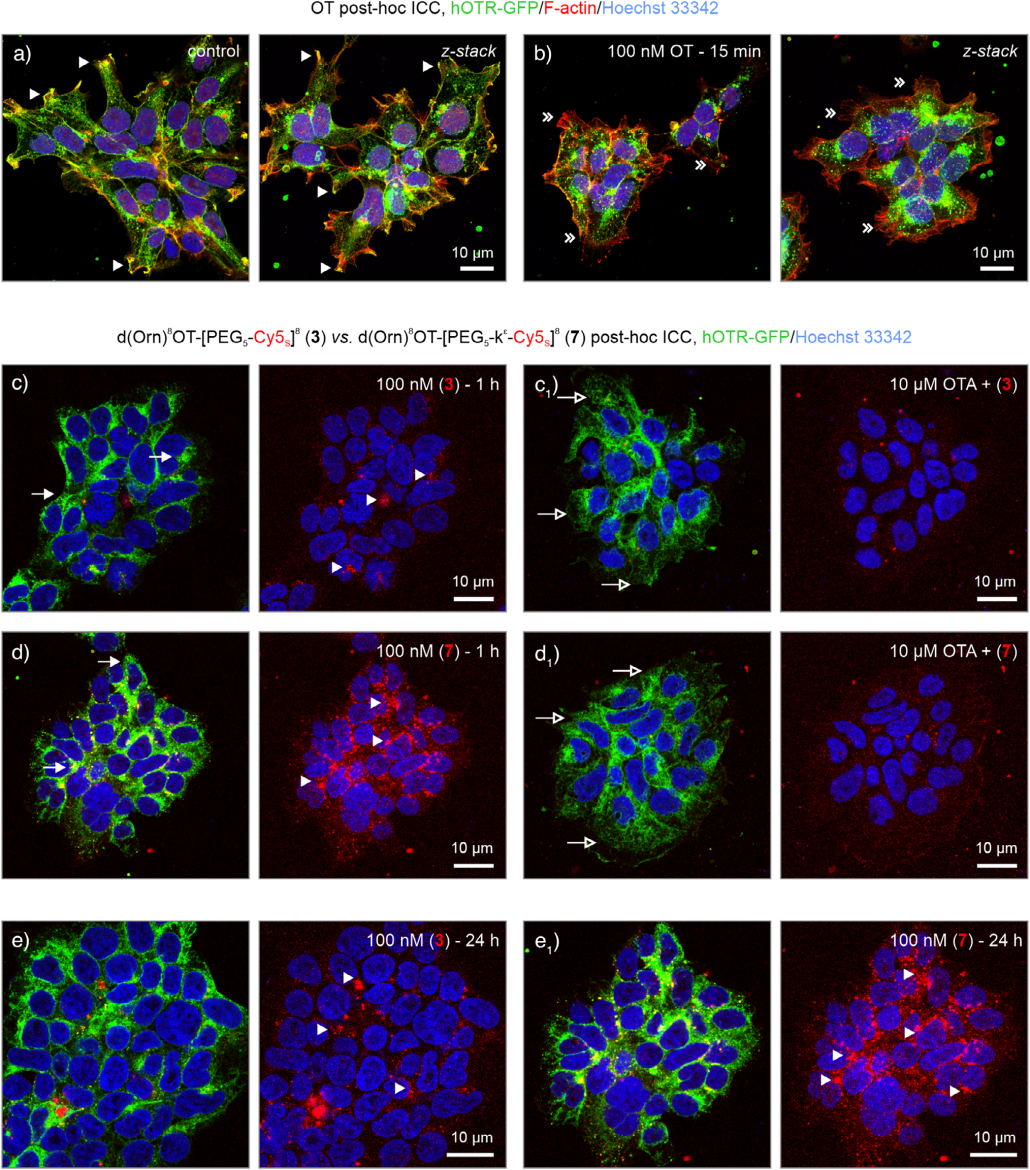
Evaluation of tracer design regarding OTR specificity and compatibility with immunocytochemistry (ICC) protocols. a) and b) OT (100 nM) potently induced internalization of hOTR‐GFP (*green*) away from the membranes into the cytoplasm (*arrowheads* versus *double arrowheads*). Note that the GFP^+^ vesicles are appearing in the cytoplasm. Z‐stacks show complete 3D images of cells reduced to a single plane. Phalloidin‐555 (F‐actin, *red*) was used to indicate membranes. c) and d) Comparison of d(Orn)^8^OT‐[PEG_5_‐Cy5_s_]^8^ (**3**) and d(Orn)^8^OT‐[PEG_5_‐k^ε^‐Cy5_s_]^8^ (**7**) revealed GFP internalization (*arrows*) after 1 h of stimulation to accrue strong signals for confocal microscopy at 100 nM and ICC protocols for both tracers. The presence of d‐Lys in d(Orn)^8^OT‐[PEG_5_‐k^ε^‐Cy5_s_]^8^ (**7**) for ICC cross‐linking with formaldehyde resulted in more retention (*arrowheads*, c versus d) than d(Orn)^8^OT‐[PEG_5_‐Cy5_s_]^8^ (**3**) lacking d‐Lys. (c_1_,d_1_) A 30‐min pretreatment with 10 µM of OTR antagonist (OTA) before 1‐h stimulation prevented GFP internalization and tracer accumulation for both tracers. Note the diffuse presence of membrane‐bound GFP signals (*open arrows*) compared to strong punctate cytoplasmic signals (*arrows*). (e,e_1_) Signal intensity was increased for compound (**7**) but not for (**3**) after prolonged stimulation (24 h), indicating that d‐Lys was required for post‐fixation ICC protocols and subsequent imaging.

To validate the application scope of our tracers for biological systems and post‐hoc imaging, we used HEK293 cells stably overexpressing human OTR or V_1a_R, tagged with a C‐terminal fused green fluorescent protein to visualize receptor trafficking (hOTR‐GFP or hV_1a_R‐GFP, respectively). Stimulation with OT and subsequent immunolabeling and confocal microscopy revealed a concentration‐dependent internalization of hOTR‐GFP, with GFP signals moving away from the cell membrane into the cytoplasm (Figure 4a,b).^[^ ^3^, ^47^ ^]^ Next, we verified the biological activity and imaging capability of tracer d(Orn)^8^OT‐[k^ε^‐k^ε^‐Cy5_s_]^8^ (**5**), lacking a PEG linker but containing d‐Lys for cross‐linking. Internalization of hOTR‐GFP and phosphorylation of cAMP‐responsive element binding protein (CREB) were observed within the same time frame as OT, in concentrations comparable with its OTR affinity (Table 1; Figure S6a–a2). Detection of tracer (**5**) overlapped with GFP signals, indicating that the tracer was co‐internalized with hOTR‐GFP. Conversely, Cy5_s_ signals were not detected in parent HEK293 cells lacking hOTR‐GFP, nor was pCREB induced, confirming that intracellular accumulation was receptor‐dependent (Figure S6b). Fluorescent signals remained visible in the cytoplasm after fixation and cytochemistry protocols (see *Materials and Methods in* Supporting Information).

To validate whether cross‐linking was required for post‐hoc visualization, we compared d(Orn)^8^OT‐[PEG_5_‐Cy5_s_]^8^ (**3**) versus d(Orn)^8^OT‐[PEG_5_‐**k^ε^**‐Cy5_s_]^8^ (**7**). Stimulation of hOTR‐GFP expressing HEK293 cells with 100 nM of tracers (**3**) and (**7**) resulted in significant hOTR‐GFP internalization (Figure 4c,d). The lack of a free amine group (d‐Lys) for crosslinking resulted in tracer (**3**) washout after ICC protocols. By contrast, tracer (**7**) remained present and detectable (Figure 4c versus Figure 4d) even after multiple 10‐min PBS washing steps and nuclear counterstaining. Pretreatment with 10 µM of the OTR peptide antagonist OTA (Table 1) prevented hOTR‐GFP internalization and cytoplasmic accumulation, confirming OTR specificity (Figure 4c_1_,d_1_). To determine if the above was (time) dependent on total tracer accumulation, we subsequently exposed our cultures to either tracer overnight. Even after 24 h of treatment, tracer (**3**) was scarcely detectable compared to (**7**) (Figure 4e,e_1_), indicating that a single free amine group was required and sufficient for formaldehyde‐based cross‐linking for post‐hoc imaging purposes.

**Figure 5:**
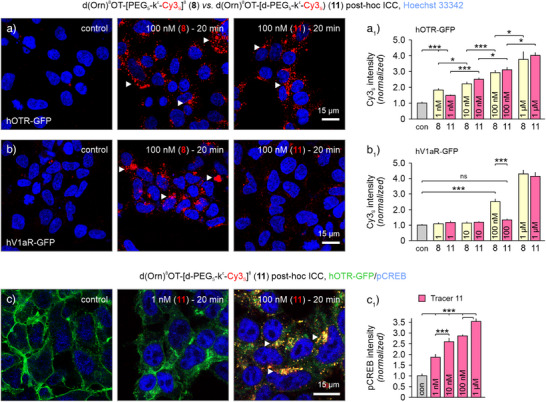
Validation of OTR selectivity and biological activity for tracers d(Orn)^8^OT‐[PEG_5_‐k^ε^‐Cy3_s_]^8^ (**8**) and d(Orn)^8^OT‐[d‐PEG_5_‐k^ε^‐Cy3_s_]^8^ (**11**). a‐b_1_) Cy3_s_ intensity measurements of both tracers in hOTR‐GFP and hV_1a_R‐GFP HEK293 cells post ICC (normalized to control). Tracers (**8**) and (**11**) displayed a significant concentration‐dependent accumulation of fluorescent Cy3_s_ (*red*) signals in hOTR‐expressing cells (a,a_1_; *arrowheads*). (b,b_1_) In hV_1a_R‐containing cells, only tracer (**8**), but not (**11**), displayed fluorescent signals and internalization at 100 nM. (b_1_) Selectivity was lost at 1 µM for both compounds, in line with the determined affinities (Table 1). *n* = 6–9 cell clusters per condition. c,c_1_) Treatment with tracer (**11**) confirmed a concentration‐dependent GFP and compound internalization (*arrowheads pointing to co‐localizing red and green puncta*). CREB phosphorylation events were observed from 1 nM onward. Data are presented as mean ± SEM (*n* = ∼150 cells over two coverslips), and statistical analysis was done applying Student's *t*‐test (*P < 0.05; ***P < 0.001; ns, not significant).

While the addition of d‐Lys improved the imaging capabilities of our tracers and did not affect hOTR binding, we observed an increase in affinity toward hV_1a_R (Table 1). We hypothesized that a negatively charged moiety, such as the carboxylic acid in the side chain of d‐Asp, could increase hOTR selectivity. Tracers d(Orn)^8^OT‐[d‐PEG_5_‐Cy5_s_]^8^ (**10**) and d(Orn)^8^OT‐[d‐PEG_5_‐k^ε^‐Cy3_s_]^8^ (**11**) indeed had increased OTR selectivity (hV_1a_R/hOTR K_i_ ratio of 92 and 114, respectively; Table 1), and in vitro, tracer (**11**) displayed a concentration‐dependent increase of bright cytoplasmic Cy3_s_ signals when presented to hOTR‐GFP cells (Figure 5a,a_1_). In line with their determined binding affinities (Table 1), d(Orn)^8^OT‐[PEG_5_‐k^ε^‐Cy3_s_]^8^ (**8**) with 15‐fold hOTR selectivity displayed significant signals in the panel of hV_1a_R‐GFP expressing cells from 100 nM onwards compared to the more selective tracer (**11**) (Figure 5b,b_1_). At micromolar concentrations, the selectivity of tracer (**11**) for hOTR was also lost (Figure 5b_1_), recapitulating our pharmacological findings. Pretreatment with 10 µM of either peptide antagonist OTA or the small molecule antagonist L368,899 (hOTR IC_50_  =  26 nM)^[^ ^48^ ^]^ completely prevented tracer (**11**) uptake (Figure S7a–a_3_). Furthermore, we generated a scrambled cyclic tracer dCINYQCP(Orn)G‐[d‐PEG_5_‐k^ε^‐Cy3_s_]^8^ (**SCR**, Table 1) based on tracer (**11**) as an additional control. Application of this scrambled tracer at 100 nM did not induce hOTR‐GFP internalization or accumulate in the cell cytoplasm (Figure S7a_4_), nor could it displace [^3^H]‐OT or [^3^H]‐VP binding at 10 µM on OTR, V_1a_R, V_1b_R, and V_2_R (Table 1, Figure S7a_5_), indicating that the linker moiety alone did not interact with the receptors.

To test whether our tracer design is compatible with a variety of structurally different fluorophores, we attached AF488 and TAMRA to our most selective peptide‐linker precursor, yielding d(Orn)^8^OT‐[d‐PEG_5_‐k^ε^‐AF488]^8^ (**12**) and d(Orn)^8^OT‐[d‐PEG_5_‐k^ε^‐TAMRA]^8^ (**13**). As compound (**13**) can be imaged with our hOTR‐GFP overexpression system, we next tested its applicability as a receptor tracer. Similar to compound (**11**), we observed a dose‐dependent uptake and GFP internalization from 10 nM onwards (Figure S8), which was completely prevented by a 30‐min pretreatment with 10 µM of OTR antagonist up to 100 nM concentrations. However, the antagonist was unable to completely block uptake at 1 µM concentrations of tracer (**13**), similar to our previous findings with compound (**11**) (Figure S8c_1_). Since the Cy3_S_‐conjugated compound appeared brighter when imaging stimulated cells and has a higher quantum yield than TAMRA,^[^ ^43^ ^]^ we continued our experimental validations below with tracer (**11**).

Next, we verified that the tracers retained biological activity at OTR by analyzing pCREB induction with a concentration range spanning from 1 nM to 1 µM of compounds d(Orn)^8^OT‐[PEG_5_‐k^ε^‐Cy3_s_]^8^ (**8**) and d(Orn)^8^OT‐[d‐PEG_5_‐k^ε^‐Cy3_s_]^8^ (**11**). We first detected similar tracer/signal accumulation and GFP internalization from 10 nM onward for both compounds (Figure 5c versus Figure S9a). Significant phosphorylation of CREB was observed starting as early as 1 nM, which tapered off after 10 nM for tracer (**8**), with (**11**) still able to increase pCREB levels up to 1 µM (Figure 5c_1_, versus Figure S9a_1_).

**Figure 6:**
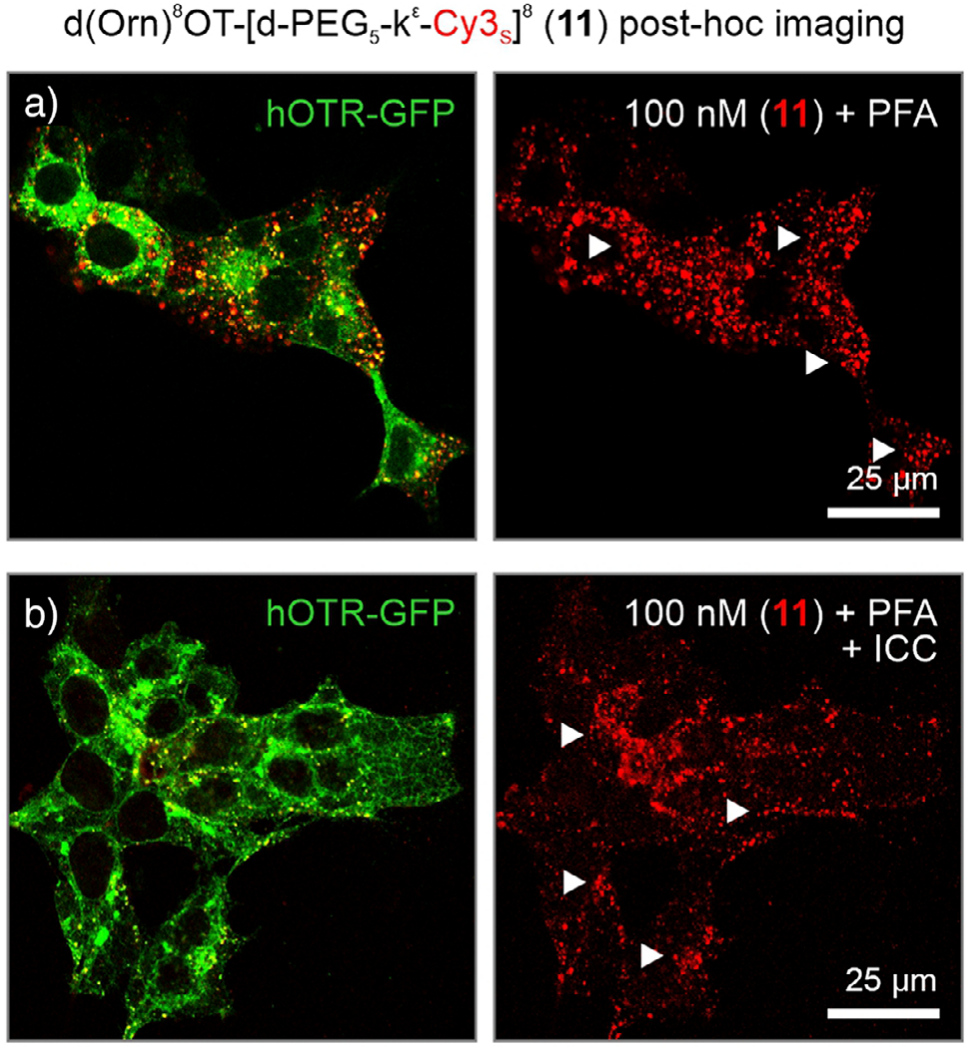
Effects of paraformaldehyde (PFA) fixation and immunocytochemistry on d(Orn)^8^OT‐[d‐PEG_5_‐k^ε^‐Cy3_s_]^8^ (**11**) detectability. a) After 20‐min stimulation and PFA fixation, a strong accumulation of tracer (**11**) was observed (*arrowheads*). b) After applying immunocytochemistry protocols, including nonspecific protein blocking, primary/secondary antibody labeling, and extensive washing steps, fluorescent intensity was reduced but still readily detectable with confocal microscopy.

Finally, we examined the post‐hoc processing effects of tracer (**11**) and observed strong Cy3_s_ signals present when imaging directly after paraformaldehyde (PFA) fixation (Figure 6a). After subsequent ICC protocols, which include antibody labeling and extensive washing steps, the number of detectable fluorophores was reduced but still visible to the naked eye. We posit that this reduction in overall fluorescence results from prolonged exposure to the detergent Triton X‐100 used for membrane permeabilization and antibody penetration (∼16 h). As such, cytoplasmic and vesicle membranes are disrupted, allowing a portion of the tracer to be washed away. However, through adjustment of laser power and gain, we could compensate for the loss of signal, resulting in robust tracer (**11**) visualization even after ICC (Figure 6b).

### Tracer Application in Live‐Cell Imaging

**Figure 7:**
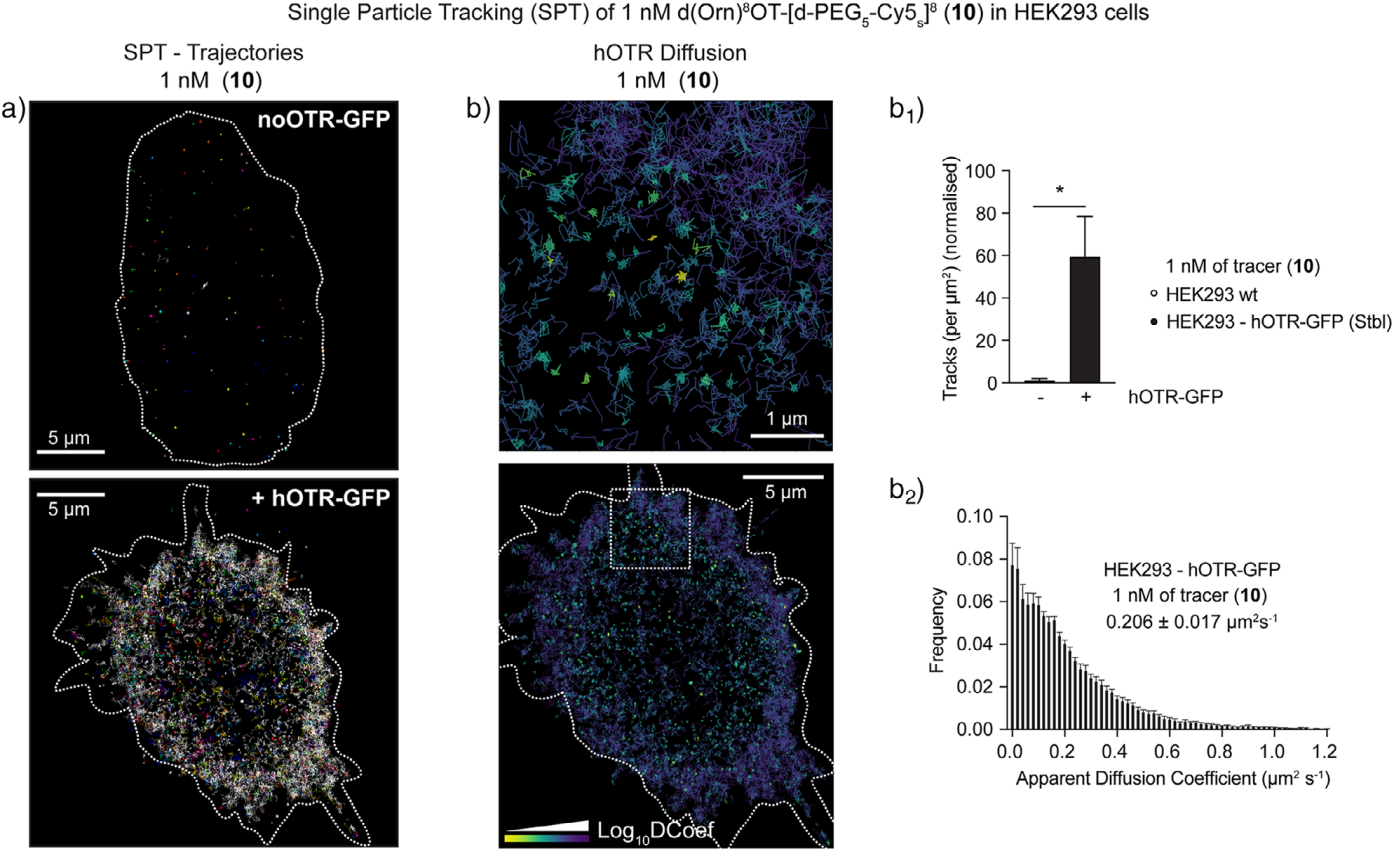
Live single‐particle tracking (SPT) of hOTR using d(Orn)^8^OT‐[d‐PEG_5_‐Cy5_s_]^8^ (**10**). HEK293 parent cells or HEK293 cells stably expressing hOTR‐GFP were imaged on glass‐bottom dishes in an isotonic imaging buffer. a) d(Orn)^8^OT‐[d‐PEG_5_‐Cy5_s_]^8^ (**10**) was applied to the dish (1 nM), and images were acquired immediately (50 Hz for 16 000 frames). Single‐molecule detections were localized and tracked using PALMtracer software. Super‐resolved trajectory images of representative cells are shown. b) A representative image of each track, pseudo‐colored by its diffusion coefficient (D) (Log_10_D, confined molecules represented by warmer colors). (b_1_) The number of tracks (per µm^2^) was calculated for both groups and normalized to the HEK293 control line. (b_2_) The apparent diffusion coefficient was calculated for all tracks in each HEK293‐hOTR‐GFP cell and plotted as a frequency distribution. Data are presented as mean ± SEM. An unpaired *t*‐test with Welch's correction was used to compare the track density (*p* < 0.05, * was considered significant).

We next tested whether d(Orn)^8^OT‐[d‐PEG_5_‐k^ε^‐Cy3_s_]^8^ (**11**) was suitable for real‐time tracking of receptor internalization and trafficking. We first exposed hOTR‐GFP cells to 10 and 100 nM of tracer (**11**) during acquisition with a ZEISS Lattice SIM microscope (Movies S1 and S2). We noticed an immediate internalization response as demonstrated by robust trafficking of both GFP and tracer (**11**). hOTR‐GFP‐positive vesicles were mobilized within seconds (Figure S10) and reached peak internalization at around 5–10 min. In addition, we noted a strong cellular contractive response typical for G_q/11_‐mediated cytoskeletal contraction through OTR activation, reconfirming maintained biological activity. To further disentangle the dynamics of our tracer‐mediated OTR engagement and trafficking, we evaluated its applicability in single‐particle tracking (SPT) in living cells. We cultured HEK293 (parent cells) and HEK293 cells stably expressing hOTR‐GFP (HEK293‐hOTR‐GFP) and performed total internal reflection microscopy (TIRFM) SPT universal point accumulation imaging in nanoscale topography (uPAINT) following the addition of 1 nM of tracer (**10**). Live SPT acquisitions (50 Hz) were performed immediately following the addition of 1 nM of (**10**) (in far red, Cy5_s_) (Movie S3). This concentration was selected to facilitate the detection and tracking of single molecules of the fluorescent agonist tracer following binding to hOTR at the plasma membrane by uPAINT. After the acquisition, the single detections of (**10**) were localized and tracked using PALMtracer (Figure 7a,b).^[^ ^49^ ^]^ We first assessed the number of tracks (indicative of the number of single molecules detected/tracked). In the absence of hOTR‐GFP expression in HEK293 cells, very few molecules were detected. By contrast, we obtained a higher density of tracks when imaging HEK293‐hOTR‐GFP cells (Figure 7a,b_1_). This supported the selectivity of the tracer to hOTR, together with the ICC findings using the equivalent compound conjugated to Cy3_s_ (**11**). We quantified the mean squared displacement of each trajectory to calculate its apparent diffusion coefficient. The diffusion coefficient of each trajectory of the tracer bound to hOTR was plotted as a frequency distribution (Figure 7b_2_). The average diffusion for tracer (**10**) was 0.206 ± 0.017 µm^2^ s^−1^. To our knowledge, this is the first assessment of single‐molecule nanoscale mobility of hOTR in living cells, demonstrating the suitability of our tracers for advanced optical methods providing unprecedented spatiotemporal resolution of OTR.

### Application of Tracer (**11**) in Fluorescence‐Activated Cell Sorting (FACS)

**Figure 8:**
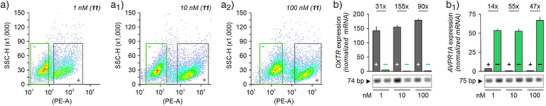
Fluorescence‐activated cell sorting (FACS) of OTR‐ and V_1a_R‐expressing HEK293 cells with tracer (**11**). a‐a_2_) FACS plots of collected cells sorted from a mixed pool of free‐floating OTR^+^ and V_1a_R^+^ cells after treatment with d(Orn)^8^OT‐[d‐PEG_5_‐k^ε^‐Cy3_s_]^8^ (**11**) at 1, 10, or 100 nM. Positive (+, *black*) and negative (‐, *green*) fractions collected were marked with a rectangle. b,b_1_) RT‐qPCR quantifications for human OTR (gene: *OXTR*) and V_1a_R (gene: *AVPR1A*) revealed substantial enrichment of OTR in the positive pool and V_1a_R in the negative pool from 1 nM onwards. Optimal separation was achieved with 10 nM of tracer (**11**) (a_1_), reflected by the highest fold enrichment of OTR (b). Values were normalized to the negative (b) and positive (b_1_) 10 nM fraction. qPCR products after 40 cycles are indicated below. Data are presented as mean ± SEM from technical triplicates.

We subsequently examined the applicability of d(Orn)^8^OT‐[d‐PEG_5_‐k^ε^‐Cy3_s_]^8^ (**11**) in advanced live‐cell applications. We pooled an equal amount of free‐floating OTR‐ and V_1a_R‐expressing HEK293 cells and incubated them for 30 min with 1–100 nM of tracer (**11**). FACS for red fluorescent signal efficiently distinguished two populations from 1 nM onward, with 10 nM giving the best separation (Figure 8a–a_2_). Subsequent real‐time quantitative PCR (RT‐qPCR) for genes *OXTR* (OTR) and *AVPR1A* (V_1a_R) confirmed an increase of OTR mRNA in the positive fraction only, with 10 nM providing the highest enrichment (Figures 8b and S11). In the negative fraction, only V_1a_R mRNA was enriched, confirming that the lead tracer was selective and could separate OTR‐ from V_1a_R‐expressing cells (Figures 8b_1_ and S11).

### Applicability of Tracer (**11**) in Primary Cell Types

**Figure 9:**
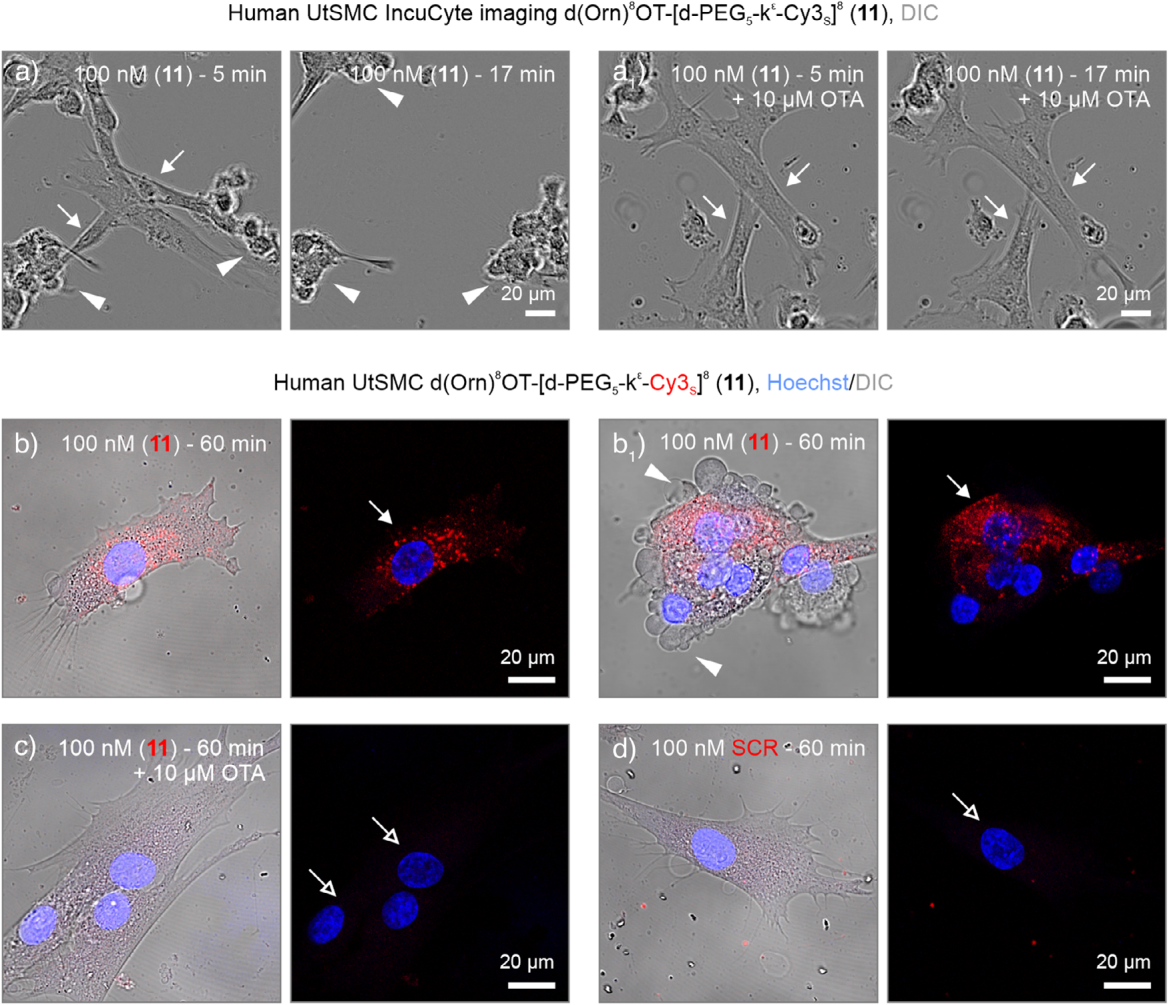
Stimulation of human uterine smooth muscle cells (UtSMC) with tracer (**11**). a,a_1_) Live‐cell imaging with an IncuCyte SX‐5 revealed rapid contraction of UtSMC cells (*arrowheads* versus *arrows*) when exposed to 100 nM tracer (**11**), which was prevented by OTA pretreatment (*t* in min). b) and c) Tracer (**11**) accumulated in vesicle‐like structures in UtSMC somata (*arrows*) but was not taken up in cells pretreated with OTA (*open arrows*). d) The sequence‐scrambled control tracer (SCR) was not internalized by the cells. Note that treatment with tracer (**11**) induced membrane blebbing (*arrowheads* in b_1_), which is associated with strong actin‐myosin‐based contractions.^[^ ^52^ ^]^ Whole cells were visualized with differential interference contrast (DIC, in left figure panels) to reveal fine details and structures.

**Figure 10:**
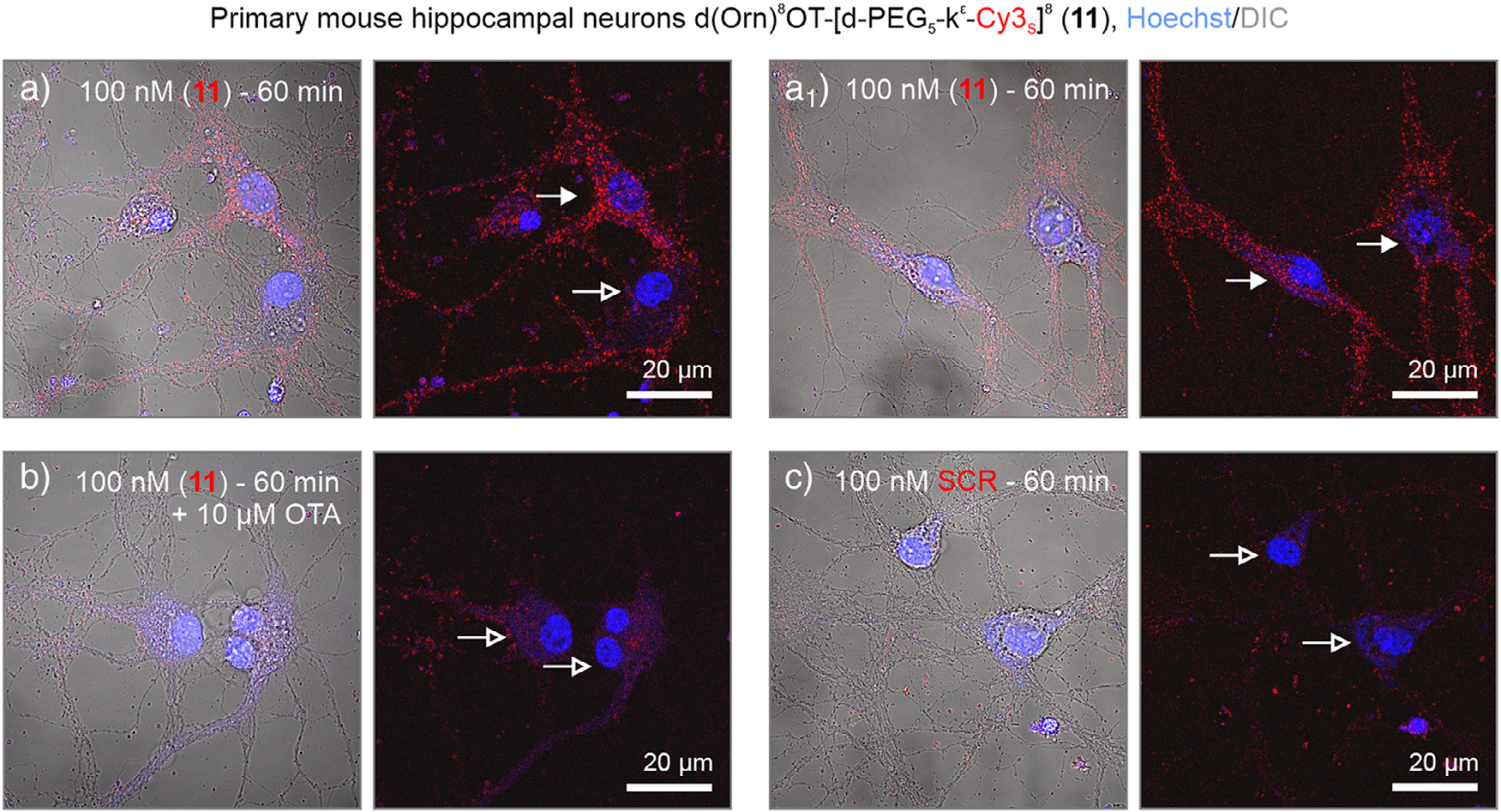
Uptake of tracer (**11**) in mouse hippocampal neurons. a,a_1_) Tracer (**11**) was internalized in a subpopulation of embryonic (E16.5) hippocampal neurons cultured for 14 days *(arrows* versus *open arrows*). b) and c) Pretreatment with mOTR‐specific OTA prevented compound (**11**) internalization, while the sequence‐scrambled control tracer (SCR) was not taken up by neurons. Whole cells were visualized with differential interference contrast (DIC, in left figure panels) to reveal fine details and structures.

Finally, we evaluated whether tracer (**11**) is applicable in primary cells endogenously expressing OTR. We first cultured human uterine smooth muscle cells (UtSMC), which respond to OT to induce uterine contractions, a key mechanism for labor.^[^ ^50^, ^51^ ^]^ Application of 100 nM compound (**11**) induced rapid contractions within minutes, which could be prevented by pretreatment with OTA (Figure 9a,a_1_). Tracer (**11**) accumulation was observed in vesicle‐like structures within 60 min, and a subpopulation of cells displayed membrane blebbing, associated with actin‐myosin‐based contractions (Figure 9b,b_1_).^[^ ^52^ ^]^ Pretreatment with OTA prevented tracer uptake as well as cellular contractions and membrane blebbing (Figure 9c,d). The scrambled tracer was not internalized by the UtSMC cells. Given the expression of functional OTRs in the rodent hippocampus,^[^ ^53^, ^54^ ^]^ we next cultured mouse and rat embryonic day (E) 16.5 hippocampal neurons for up to 14 days to ensure mature neurons.^[^ ^55^ ^]^ Application of 100 nM of tracer (**11**) for 1 h resulted in robust uptake in subpopulations of neurons in both mouse and rat preparations (Figures 10a,a_1_ and S12a,a_1_). Pretreatment with OTA (10 µM; 30 min) completely inhibited the uptake of tracer (**11**), while the scrambled control was inactive on cultured neurons (Figures 10b,c and S12b,c).

Taken together, our tracer design and SAR study delivered not only new bioactive and OTR‐selective fluorescent tracers but also revealed a novel design strategy to enhance OTR selectivity over VPRs by introducing a negative charge at position 8. Our lead OTR tracers are bright, selective, water‐soluble, and compatible with live‐cell and post‐hoc imaging in both overexpression and primary cell systems, thereby suitable for a broad range of sophisticated experimental applications and representing highly valuable new reporter tools for studying the OTR system.

## Discussion

Fluorescent tracers are powerful and versatile tools to study endogenous receptors and proteins and are used in numerous applications.^[^ ^20^ ^]^ Unlike approaches that involve genetic manipulation (e.g., tagging with fluorescent proteins), the application of labeled tracers preserves native protein functions and avoids artifacts such as altered expression levels, mislocalizations, and differences in pharmacological properties.^[^ ^21^, ^27^, ^56^ ^]^ Moreover, such tracers can be applied within the context of tissues and other biological samples, where the success of genome modifications can vary widely and might not be accessible.^[^ ^57^ ^]^ Fluorescent tracers also offer clear advantages over methods that rely on measuring mRNA levels, which do not necessarily correlate with protein levels and do not provide insights into the functional states of the receptor.^[^ ^58^, ^59^ ^]^ Studying proteins through selective tracers provides a more direct and accurate representation of their role under various physiological and pathological conditions. This is particularly important for receptor systems that do not have selective or reliable antibodies, which are still the gold standard for studying proteins yet notoriously difficult to produce for GPCRs.^[^ ^60^ ^]^ Importantly, the application scope of probes, such as the bioactive and target‐selective fluorescent tracers developed in this work, goes beyond the traditional antibody alternative by not only visualizing the receptor but also providing valuable insights into receptor activation, signaling, internalization, and trafficking. Recent advances in the field demonstrated the importance of spatially encoded GPCR signaling,^[^ ^61^ ^]^ and the application of fluorescent probes with well‐characterized receptor pharmacology offers the unique possibility to unravel the correlations between specific signaling pathways, membrane organization, endocytosis, and intracellular trafficking. Hence, such bioactive fluorescent tracers are expected to significantly advance our understanding of GPCR‐mediated cellular processes.

In this work, we developed bioactive and OTR‐selective tracers and characterized them across various applications, supporting the study of OTR in its endogenous state. OTR is a GPCR involved in many physiological processes with an underexplored therapeutic scope in an array of disorders, including cancer, cardiovascular diseases, diabetes, pain, digestive, metabolic, and neuropsychiatric disorders (anxiety, autism spectrum disorder, schizophrenia, etc.).^[^ ^6^, ^7^, ^8^, ^9^, ^10^, ^11^ ^]^ Although OTR research revealed important insights on biased signaling,^[^ ^62^, ^63^, ^64^ ^]^ allosteric modulation,^[^ ^29^, ^30^ ^]^ signaling dependence on subcellular localization,^[^ ^46^, ^65^ ^]^ and receptor hetero‐ and homodimerization,^[^ ^24^, ^66^, ^67^, ^68^ ^]^ we still lack a full understanding of its role within the native context of different tissues.^[^ ^4^ ^]^ To identify whether OTR or closely related VPRs are involved in certain processes, how biological outcomes are regulated on a molecular level, and which dysregulations play into the formation of specific diseases requires the application of reliable, well‐characterized, and selective reporter tools. Such molecular reporter tools are particularly scarce for OTR and VPRs, yet important for drug target validation. Biological applications remain a contentious issue due to the complex chemical composition and molecular interactions in living and post‐fixed tissues. Therefore, we took a broad range of factors into account when designing our fluorescent probes, ranging from handling ease, pH dependence, photostability, water‐solubility, fixability, metabolic stability, signal brightness and wavelengths, target and off‐target pharmacology, and various application protocols. With d(Orn)^8^OT as our starting point, we strategically focused on the linker connecting the peptide to the fluorophore as a site for modulating selectivity. This approach was driven by the cryo‐EM structure of the OT‐OTR complex, revealing binding interactions of every peptide residue and the well‐documented sensitivity of the OT‐OTR binding pocket to even minor structural modifications (e.g., ring size modification, disulfide‐to‐dicarba substitution, tyrosine‐OH alkylation, and C‐terminal deamidation all affect binding and selectivity). By contrast, we hypothesized that the linker, which protrudes outside the binding pocket, provides a handle to influence receptor selectivity without compromising affinity through engaging with parts of the receptor that are not crucial for binding. Hence, we explored various linker designs to pursue tracer applicability as well as OTR selectivity. PEG spacers were chosen due to good water solubility, chemical inertness, metabolic stability, and lack of toxicity or immune response, which is important for future in vivo applications. The introduction of metabolically stable d‐Lys enabled efficient cross‐linking for ICC applications while avoiding undesired proteolytic linker cleavage. The lack of OTR selectivity of parent peptide d(Orn)^8^OT and analogs carrying d‐Lys close to residue 8 prompted us to test whether selectivity can be induced by incorporating a negative charge, particularly considering that VP has a positively charged arginine in position 8, which is important for VPR binding and activation. This strategy worked well, and introducing negatively charged d‐Asp into the linker design was critical in boosting OTR selectivity >100‐fold against its closely related VPRs. We carried out molecular docking studies to identify receptor residues responsible for the observed selectivity shift; however, while the docking approach worked well for OT and d(Orn)^8^OT, it did not provide feasible results for the structurally more complex tracers, likely due to the conformational flexibility and water‐engaging properties of the PEG spacer groups that are difficult to model. For imaging purposes, both Cy3_s_ and Cy5_s_ turned out to be reliable fluorophores due to their pH stability, water solubility, photostability, and compatibility for high signal‐to‐noise ratio, high‐resolution single‐molecule labeling in microscopy and cytochemistry, FRET,^[^ ^69^ ^]^ and microarrays.^[^ ^70^ ^]^ Due to their distant peak excitation (Cy3_s_, 550 nm; Cy5_s_, 650 nm) and emission wavelengths (Cy3_s_, 570 nm; Cy5_s_, 670 nm), they can also be used for dual labeling. While Cy5_s_ is excited at a longer wavelength, leading to less autofluorescence and increased signal‐to‐noise ratio, Cy3_s_ has the advantage of being observed with the naked eye and being suitable for epifluorescent microscopy. Importantly, the tracers retained their pharmacological and biological profiles independent of the type of fluorophores attached (Cy3_s_, Cy5_s_, AF488, TAMRA), confirming that the linker design is the main contributor to OTR selectivity. Taken together, our tracers represent powerful new tools for studying OTR expression and signaling. They are broadly compatible with a range of advanced techniques, including confocal and super‐resolution microscopy, ICC, SPT, FACS, and receptor detection in primary cells. By applying these novel neuropeptide probes, we aim to deepen our understanding of the complex roles of OTR in health and disease. Furthermore, extending this approach to other peptide GPCRs may enable the development of a new class of reporter tools.

## Conclusions

Our efforts yielded a new generation of potent OTR tracers unique in their OTR selectivity, biological activity, water solubility, brightness, and fixability for post‐hoc imaging, rendering them versatile tools for a wide array of biological applications. The successful implementation of these tracers in FACS, confocal microscopy, and SPT in both overexpression and primary cell systems underscores their broad utility. Additionally, the systematic SAR study provided new insights into OTR and VPR binding, revealing a ligand selectivity switch that holds promise for future OTR ligand design. These innovative tracers are poised to become valuable tools for investigating OTR expression and signaling, with significant implications for advancing our understanding of OTR's roles in health and disease. Importantly, our approaches and design strategies are also applicable to other GPCR systems that lack reliable reporter probes.

## Supporting Information

The authors have cited additional references within the Supporting Information.^[^ ^71^, ^72^, ^73^, ^74^, ^75^, ^76^, ^77^, ^78^, ^79^, ^80^, ^81^ ^]^

## Author Contributions

M.P.B., P.K., E.K., T.L., R.G., M.Z., T.K., A.W., and M.J. designed and performed experiments. F.M., C.W.G., E.K., and M.M. supervised students and guided experimental design. M.P.B., P.K., E.K., R.G., T.L., and M.M. wrote the manuscript.

## Conflict of Interests

The tracers developed are patented. All authors have read, commented on, and approved the manuscript and have not expressed any further conflicts of interest.

## Supporting information



Supporting Information

Supporting Information

Supporting Information

Supporting Information

## Data Availability

The data that support the findings of this study are available in the Supporting Information of this article.
