# Supplementary material for: Fluorescent Peptide Tracers for Simultaneous Oxytocin Receptor Activation and Visualization
Source: Angew Chem Int Ed Engl. 2025 Sep 26;64(46):e202515180. doi: 10.1002/anie.202515180 (PMC12603982; doi:10.1002/anie.202515180)
Supplement: Supplementary file 1 — Supporting Information [file ANIE-64-e202515180-s001.pdf]

Supporting Information for

Fluorescent peptide tracers for simultaneous oxytocin receptor activation and visualization

Monika Perisic Böhm[1,2], Predrag Kalaba[1], Rachel S. Gormal[3], Maja Zupančič[4], Alexandra Wolf[4], Mia Juračić[1], Thomas Kremsmayr[1], Frédéric A. Meunier[3,5], Thierry Langer[6], Christian W. Gruber[7], Erik Keimpema[4], Markus Muttenthaler[1,8]

[1]Institute of Biological Chemistry, Faculty of Chemistry, University of Vienna, Vienna, Austria

[2]Vienna Doctoral School in Chemistry, University of Vienna, Vienna, Austria

[3]Clem Jones Centre for Ageing Dementia Research, Queensland Brain Institute, The University of Queensland, Brisbane, Australia

[4]Department of Molecular Neurosciences, Center for Brain Research, Medical University of Vienna, Vienna, Austria

[5]School of Biomedical Sciences, The University of Queensland, Brisbane, Australia

[6]Department of Pharmaceutical Chemistry, Faculty of Life Sciences, University of Vienna, Vienna, Austria

[7]Institute of Pharmacology, Center for Physiology and Pharmacology, Medical University of Vienna, Vienna, Austria

[8]Institute for Molecular Bioscience, The University of Queensland, Brisbane, Australia

This document includes:

I. Experimental Section 3
II. Supporting Tables 9
III. Supporting Figures 10
IV. Supporting Movies 26
V. References 27

I. Experimental Section

Ethical Considerations. Pregnant C57BL/6JRj mice and Wistar rats (Janvier) were kept under standard housing conditions with a 12/12h dark/light cycle in humidity and temperature-controlled rooms until ready for embryo harvesting. Food and water were provided *ad libitum*. Tissue collection was approved by the Austrian Federal Ministry for Women, Science and Research (2024-0.518.937).

Materials. Solvents, reagents, media, as well as antibiotics and radioligands, were purchased from commercial suppliers and applied without further purification. Ultrapure water (Milli-Q®, ddH$_2$O) was used for all aqueous solutions and buffer preparations. No unexpected or unusually high safety hazards were encountered.

Synthesis. Fmoc-protected L- and D-amino acids were purchased from Sigma-Aldrich, Merck (Asn, Cys, Gln, Gly, Ile, Orn, Pro, Tyr), and Iris Biotech (Asp, Lys). Desamino building blocks were purchased from Bachem (3-mercapto-3,3-cyclopentamethylenepropionic acid (d(CH$_2$)$_5$)) and Iris Biotech (mercaptopropionic acid (Mpa)). Fmoc-PEG$_5$-OH was purchased from Iris Biotech GmbH, and fluorophores from Cayman Chemical (Cy3$_s$-NHS), Combi-Blocks (Cy5$_s$-NHS), Lumiprobe (Alexa Fluor 488 NHS (AF488-NHS)), and Szabo Scandic (5-TAMRA-NHS). Solid-phase peptide synthesis (SPPS) reagents and solvents were purchased from Sigma-Aldrich, Merck (acetic anhydride, ascorbic acid, iodine, 4-dimethylaminopyridine (DMAP), *N,N'*-diisopropylcarbodiimide (DIC), *N*-hydroxysuccinimide (NHS), *N,N'*-diisopropylethylamine (DIPEA), Oxyma Pure, piperidine, pyridine, triisopropylsilane (TIPS)), VWR Chemicals (diethyl ether, dimethylformamide (DMF)), Apollo Scientific (HATU), and Thermo Fisher Scientific (dichloromethane (DCM)). Fmoc-Rink amide aminomethyl (AM) resin (100-200 mesh, loading: 0.74 mmol/g, 1% divinylbenzene (DVB) cross-linking) and Wang resin (100-200 mesh, loading: 0.68 mmol/g, 1% DVB cross-linking) were purchased from Iris Biotech GmbH.

RP-HPLC(-MS) analysis and purification. Solvents and additives were purchased from VWR Chemicals (acetonitrile (ACN), trifluoroacetic acid (TFA), and formic acid (FA)), Sigma Aldrich, Merck (2-propanol), and Honeywell (methanol).

Cell culture and pharmacological assays. Dulbecco's Modified Eagle Medium (DMEM) high glucose was purchased from Thermo Fisher Scientific. Fetal bovine serum (FBS), G-418 disulfate salt powder, penicillin-streptomycin (P/S), phosphate buffer saline (PBS) tablets, and trypsin-EDTA were purchased from Sigma-Aldrich, Merck. For membrane preparation, albumin standard 2 mg/mL, Pierce bicinchoninic acid protein assay kit (BCA protein assay kit), and Pierce protease inhibitor tablets, EDTA-free, were purchased from Thermo Fisher Scientific, and a cell scraper 25 cm from Sarstedt. For binding assays, glass microfiber filters (MGB grade, 140 g/m^2) were purchased from Sartorius Stedim, magnesium chloride hexahydrate from Sigma-Aldrich, Merck, polyethyleneimine from Honeywell, and Rotiszint® eco plus LSC Universalcocktail and tris(hydroxymethyl)aminomethane hydrochloride (TRIS hydrochloride) were purchased from Carl Roth. Radioligands [^{3}H]-OT (oxytocin [tyrosyl-3,5-^{3}H]) and [^{3}H]-VP (vasopressin [phenylalanyl-3,4,5-^{3}H(N)]) were purchased from PerkinElmer. Polyplus jetPRIME transfection reagent was purchased from Sartorius.

General synthetic procedure and purification. Control peptides (OT, VP, OTA, SCR) and peptide-linker scaffolds for tracers were prepared at 0.1 mmol scale, applying the established manual Fmoc-SPPS protocol.[1] Labeling group attachment to peptide-linker scaffolds was carried out in solution after peptide cleavage, oxidative folding, and purification. All synthetic steps were performed at room temperature (25°C).

Peptide synthesis. Resin beads were swollen overnight in DMF. Rink amide AM resin was used for all peptides except for OTA. To obtain the C-terminal carboxylic acid required for OTA, a Wang resin was used and loaded manually with the first amino acid. Attachment of 4 equiv. of the C-terminal amino acid to the swollen Wang resin

was carried out overnight by applying Oxyma Pure (4 equiv.), DIC (4 equiv.), and DMAP (0.1 equiv.) activation. The effective resin loading was determined *via* photometric Fmoc quantification. 10 mg of dried resin was incubated for 30 min in 10 mL of a 20% piperidine in DMF solution. Next, the supernatant was diluted 10-fold with 20% piperidine in DMF, and absorption at 301 nm (A_{301}, absorption of the dibenzofulvene piperidine adduct, ε_{adduct} = 7,800 $cm^{-1}M^{-1}$) was measured against a 20% piperidine in DMF blank. The resin loading was obtained using a calculation based on Lambert Beer's law: resin loading (mmol/g) = A_{301}/78 x 100.[2,3] A capping step was conducted (2 equiv. acetic anhydride and 2 equiv. pyridine). Remaining amino acid couplings were carried out in the same way as with the Rink amide AM resin using HATU activation (4 eq. amino acid, 3.9 eq. HATU, 4.8 eq DIPEA) and a coupling time of 15 min, followed by 1 min DMF flow-wash, 2 x 1-min Fmoc-deprotection (50% piperidine in DMF), and a 1-min DMF flow-wash. The orthogonal Mtt protection group was removed with 3% TIPS and 1% TFA in DCM. Upon completion of peptide assembly, the resin was flow-washed with DCM for 1 min and dried under reduced pressure overnight. Peptides were cleaved and deprotected using a cleavage and global side chain deprotection cocktail (90% TFA, 5% TIPS, 5% ddH_2O; ~5 mL/0.1 mmol peptide) and a cleavage time of 2 h. The cleavage solution was collected, and the TFA evaporated under a nitrogen stream, followed by peptide precipitation with ice-cold diethyl ether and centrifugation (10 min, 3000 *g*, 4°C).

Oxidative folding. Peptides were dissolved in solvent A (ddH_2O + 0.1% TFA) under the addition of solvent B (ACN + 0.08% TFA) as required to a maximum peptide concentration of 400 μM. A 60 mM solution of iodine in methanol was added dropwise until a slightly yellow color of the solution remained. After 1 min, the excess iodine was quenched with ascorbic acid.

Purification. Following synthesis, cleavage, and folding, peptides were purified *via* RP-HPLC on a Waters Auto Purification HPLC-UV system. Depending on the amount of crude product, either a preparative (20-100 mg, Kromasil Classic C_{18} 21.2 x 250 mm, 300 Å, 10 μm) or a semipreparative (1-20 mg, Kromasil Classic C_{18} 10 x 250 mm, 300 Å, 10 μm) column was used at a solvent flow of 20 mL/min or 10 mL/min, respectively. Solvents A and B were used as eluents at a linear standard gradient of 5-45% B in 50 min. The starting concentration of B was adjusted according to the hydrophobicity of the peptide. Fractions were collected based on UV absorption at 214 nm.

Solution-phase fluorophore attachment. Peptides were dissolved in DMF (~50 mM) and 1.1 equiv. of NHS ester-activated fluorophore and 3 equiv. of DIPEA were added. Reactivation of the hydrolyzed NHS ester was carried out *in situ* under the addition of 3 equiv. NHS, DIC, and DIPEA (only with peptides that did not contain a carboxylic acid).[4] In the case of Fmoc-containing peptides, 60 equiv. of piperidine were added once the fluorophore coupling was complete, followed by piperidine quenching with TFA after 1 min. A second purification was carried out after fluorophore attachment. For the final results, see **Table S1**.

Reaction monitoring and quality control. Reactions were monitored by analytical RP-HPLC-UV-MS on a Thermo Fisher Scientific Dionex Ultimate 3000 system equipped with a Waters XSelect CSH UPLC C_{18} XP column (3.0 x 75 mm, 130 Å, 2.5 μm), UV detector (measurement at 214 nm and 280 nm), and Thermo Fisher Scientific MSQ Plus ESI-MS unit (positive ionization mode). Solvents A and B were used at a gradient of 1-61% solvent B in 6 min for hydrophilic peptides or 10-85% solvent B in 7 min for hydrophobic peptides. The flow rate was 1 mL/min.

Analytic RP-HPLC. Chromatograms for determination of product purity were recorded on a Thermo Fisher Scientific Vanquish Horizon UHPLC system using a Kromasil Classic C_{18} column (4.6 x 150 mm, 300 Å, 5 μm), a linear gradient of 5-65% solvent B in 30 min (solvent A: ddH_2O + 0.1 % TFA; solvent B: ACN + 0.08 % TFA), 1 mL/min solvent flow, and UV detection at 214 and 280 nm (**Figure S3**). Compound purity was determined through analytical RP-HPLC peak integration at 214 nm.

Final product identity. Final products were confirmed through HR-ESI-MS on a maXis HD ESI-Qq-TOF mass spectrometer (Bruker Daltonics). Samples were dissolved to 20 µg/mL in MeOH and directly infused into the ESI source at a flow rate of 3 µL/min with a syringe pump. The ESI ion source was operated as follows: capillary voltage: 0.9 to 4.0 kV (individually optimized), nebulizer: 0.4 bar (N_2), dry gas flow: 4 L/min (N_2), and dry temperature: 200°C. Mass spectra were recorded in the range of *m/z* 50-1550 in the positive-ion mode. The sum formulas were determined using Bruker Compass DataAnalysis 4.2 based on the mass accuracy ($\Delta m/z \leq 2$ ppm) and isotopic pattern matching (SmartFormula algorithm).

Determination of peptide content and concentration. Since purified and lyophilized peptides contain variable amounts of water and salts (10–70%) it is important to determine the accurate peptide concentration to support reliable pharmacological characterization. This is done *via* a well-established quality control (QC) and concentration determination procedure.[5] Ligands were dissolved in ddH_2O or a mixture of ddH_2O and ACN at 3 mg/mL. To 5 µL of this stock solution 135 µL of ddH_2O was added. 10 µL of the final solution were analyzed by RP-HPLC (n = 3) using a designated QC column (Kromasil Classic C_{18}, 100 x 2.1 mm, 100 Å, 5 µm). A solvent gradient of 5-65% B in 10 min and a flow rate of 1 mL/min were applied. The peptide concentration was calculated applying **Equations 1** and **2**, which are based on the absorption of light at 214 nm by amide bonds.[6] The absorption peak area of the peptide of interest was then compared to the absorption peak area of three standards of similar peptides (OT, CYIQNCPLG; carbetocin, (butyryl)Y^{Me}IQNCPLG; and antagonist CYVQNCPPG) with known concentration (determined by amino acid analysis). For fluorescent compounds, the contribution of the dye to absorption at 214 nm was accounted for by replacing two non-absorbing amino acids (i.e., any residue beside N, Q, Y, F, H, and W) with two tryptophan (W). For results see **Table S1**.

$$\varepsilon_{214\,nm} = (n_{AA} - 1 + n_N + n_Q + \gamma) * 2846 + n_F * 7200 + n_H * 6309 + n_W * 22735 + n_Y * 5755 \quad \textbf{(1)}$$

$\varepsilon_{214\,nm}$ … extinction coefficient of the peptide at 214 nm [$M^{-1}cm^{-1}$]

n_{AA} … total number of amino acids in the sequence

n_x … number of asparagine (N), glutamine (Q), phenylalanine (F), histidine (H), tryptophan (W), and tyrosine (Y) residues

γ … accounting for C-terminus; γ = 1 if the peptide contains a C-terminal amide, γ = 0 if the peptide contains a C-terminal carboxylic acid

$$c_{sample} = \frac{\varepsilon_{214\,nm}^{STD}}{\varepsilon_{214\,nm}^{sample}} * \frac{a_{sample}}{a_{STD}} * c_{STD} \quad \textbf{(2)}$$

c_{sample} … concentration of the peptide stock solution [µM]

c_{STD} … concentration of the peptide standard [µM]

$\varepsilon_{214\,nm}^{sample}$ … extinction coefficient of the peptide of interest at 214 nm [$M^{-1}*cm^{-1}$]

$\varepsilon_{214\,nm}^{STD}$ … extinction coefficient of the standard peptide at 214 nm [$M^{-1}*cm^{-1}$]

a_{sample} … mean peak area at 214 nm per µL injection volume of the sample; $a = \frac{mean\,peak\,area}{10}$ [mAU*min*μL^{-1}]

a_{STD} … mean peak area at 214 nm per µL injection volume of the standard; $a = \frac{\text{mean peak area}}{10}$ [mAU*min*μL^{-1}]

***In silico* docking experiments**. Docking studies were performed with OT and d(Orn)[8]OT (**1**), d(Orn)[8]OT-[PEG$_5$-k^ε-Cy3$_s$][8] (**8**), and d(Orn)[8]OT-[d-PEG$_5$-k^ε-Cy3$_s$][8] (**11**) using the AutoDock Vina 1.1 program[7] implemented in LigandScout[8]. As the protein template structure, the cryo-EM structure of OT in OTR (PDB 7RYC)[9] was used with default docking parameters starting from ligand structures in stereospecific SMILES notation.

Membrane preparation for radioligand assays. HEK293 cells were used for stable expression of GFP-tagged human OTR, $V_{1a}R$, $V_{1b}R$, and V_2R isoforms and transient expression of murine OTR, $V_{1a}R$, $V_{1b}R$, and V_2R isoforms. Cell culture work was performed under sterile conditions with DMEM high glucose containing 10% FBS, and 50 U/mL P/S. Cells were cultured in 10 cm diameter cell culture dishes at 37°C in a humidified atmosphere with 5% CO_2, and cells were passaged at 70-90% confluency. G-418 sulfate was added to the growth medium of stable cell lines at 0.5 mg/mL to sustain selection pressure, and cells were harvested at 90% confluency for membrane preparation. For transient expression of murine receptors, 60% confluent wild-type HEK293 cells were transfected using the jetPRIME transfection reagent according to the manufacturer's protocol and harvested 48 h post-transfection. For membrane preparation, cells were washed with ice-cold PBS pH 7.4, scraped into fresh PBS on ice, and centrifuged at 4°C and 178 *g* for 10 min. The pellet was resuspended in a TRIS buffer solution (5 mM $MgCl_2$, 50 mM TRIS, 0.1% BSA, pH 7.4) with protease inhibitor and disrupted three times for 10 s in an ice-cold water bath sonicator. The obtained solution was centrifuged for 20 min at 54,192 *g* and 4°C. The obtained pellet was resuspended in TRIS buffer without protease inhibitors, homogenized with an ultrasonicator, aliquoted, and stored at -80°C until further use. A BCA assay was used to determine the protein concentration of the membrane preparations.

Radioligand displacement and saturation experiments. Assay solutions consisting of competing ligand, radioligand, and membrane preparation were incubated for 1 h at 37°C. Standard binding buffer solution (50 mM TRIS, 5 mM $MgCl_2$, 0.1% BSA, pH 7.4) was used to prepare all dilutions; the final assay volume was 200 µL. Depending on the receptor, either [^{3}H]-OT or [^{3}H]-VP radioligands were applied. Applied membrane concentrations were 8 µg/vial for hOTR, 5 µg/vial for $hV_{1a}R$, and 3 µg/vial for $hV_{1b}R$ and hV_2R, respectively, and 4 µg/vial for mOTR, 1.5 µg/vial for $mV_{1a}R$, 3 µg/vial for $mV_{1b}R$, and 10 µg/vial for mV_2R. To assess non-specific binding, 10 µM of either OT or VP was added instead of the test ligand. Specific binding was determined by subtraction of the non-specific binding from the total binding. In radioligand displacement experiments, semi-logarithmically spaced concentrations of respective test ligands were applied to determine K_i values based on assessed K_d values of radiotracers and by fitting the data to a three-parameter logistic Hill equation and applying the Cheng and Prusoff approximation.[10] In case of human receptors from stable cell lines, K_d and B_{max} values were obtained from saturation binding experiments and were 0.65 nM and 4.2 pmol/mg for [^{3}H]-OT at hOTR, 0.21 nM and 2.6 pmol/mg for [^{3}H]-VP at $hV_{1a}R$, 0.14 nM and 2.9 pmol/mg for [^{3}H]-VP at $hV_{1b}R$, and 2.95 nM and 25.5 pmol/mg for [^{3}H]-VP at hV_2R, respectively (**Figure S4**). In case of murine receptors from transient expression, K_d values were taken from the literature and were 0.54 nM for mOTR, 1.3 nM for $mV_{1a}R$, 0.67 nM for $mV_{1b}R$, and 0.4 nM for mV_2R.[11,12] 100 % binding at murine receptors corresponds to 2.4 pmol/mg, 6.6 pmol/mg, 1.8 pmol/mg, and 0.9 pmol/mg for mOTR, $mV_{1a}R$, $mV_{1b}R$, and mV_2R, respectively. Data analysis was performed using GraphPad Prism (version 9.5.1). After incubation, membranes were filtered and washed on a Skatron Cell Harvester using polyethyleneimine-coated glass fiber filters (type B, soaked in 0.1% aqueous polyethyleneimine solution for 15 min and dried) by applying three wash cycles with wash buffer (10 mM TRIS, 1 mM $MgCl_2$, pH 7.4, 4°C). The soaked filter pieces were transferred into counter vials, and 2 mL of scintillation cocktail (LSC Universalcocktail) was added to each vial. After 1 h incubation at 25°C on a shaker, radioligand concentration was determined using a Tri-Carb liquid scintillation analyzer.

***In vitro* phosphorylation assay.** Stably expressing hOTR-GFP and $hV_{1a}R$-GFP HEK293 cells were grown in DMEM, 10% FBS, 1 mM sodium pyruvate, and P/S or G-418 (500 µg/mL, all from Thermo Fisher Scientific), respectively, at 37°C and 5% CO_2. For experiments, trypsin (0.1%, Thermo Fisher Scientific) dissociated cells were plated on poly-D-lysine-coated glass coverslips at a 50,000 cells/well density and grown overnight in a full growth medium. The next day, the medium was replaced with a serum-free medium for cell phase synchronization and increased sensitivity to external stimuli. Cells were treated with concentrations ranging from 1 nM to 1 µM of OT or conjugated compounds up to 24 h (n = 2 coverslips per condition), washed, and fixed in 4% PFA in PBS (Sigma-Aldrich), including PhosSTOP (Hoffmann-La Roche) phosphatase inhibitors, for 30 min. Coverslips were subsequently washed with PBS and processed for immunocytochemistry.

Immunocytochemistry and imaging. Unspecific protein binding was quenched with 5% normal donkey serum (Jackson ImmunoResearch), 2% bovine serum albumin (Sigma-Aldrich, Merck), and 0.2% Triton X-100 (Sigma-Aldrich, Merck) in PBS. Next, cells were exposed to primary antibody cocktails containing FITC-conjugated goat-anti-GF (1:2000, Sigma-Aldrich, Merck) and mouse pCREB (1:300, Sigma-Aldrich, Merck) in PBS with 2% normal donkey serum, 0.1% BSA, and 0.2% Triton X-100 overnight at 4°C. After three PBS washing steps, wells were incubated with secondary antibody cocktails (1:400, Jackson Immuno Research) containing suitable conjugated fluorophores, as well as Alexa Fluor 555/647 Phalloidin (1:500, Thermo Fisher Scientific) and Hoechst 33342 (1:5,000; Sigma-Aldrich, Merck) as cytoskeletal and nuclear counterstain, respectively. Cells and tissues were mounted with glycerol gelatin (Sigma-Aldrich, Merck) and imaged on a Zeiss LSM 780/880 confocal microscope (Zeiss). Images were analyzed and equally contrasted for visualization with ZEN Pro (Zeiss) and collated in CorelDRAW 2019.

Live cell imaging. For 3D live cell imaging, attached and serum-starved HEK293-hOTR-GFP cells in 96-well glass bottom plates were imaged on a ZEISS Lattice SIM Microscope using a 40x oil objective and SIM^2 post-processing (*standard*). Whole *z*-stacks were collapsed and combined for **Movies S1** and **S2**, as well as **Figure S10**. For SPT of tracer (**10**) in HEK293 (parent cell line) and HEK293-hOTR-GFP cells by TIRF microscopy, cells were visualized on a TIRF microscope equipped with an $iLas^2$ double laser illuminator (Roper Scientific), a CFI Apo TIRF 100×/1.49 NA oil-immersion objective (Nikon), and an Evolve 512 Delta EMCCD camera (Photometrics) mounted on a TwinCam LS Image Splitter (Cairn Research), a Perfect Focus System (Nikon), an $iLas^2$ double-laser illuminator (Roper Scientific) for 360° TIRF illumination, a 642 nm laser (100 mW, Vortran), a double beam splitter (LF488/561-A-000, Semrock) and double band emitter (FF01-523/610-25, Semrock). Images were acquired at 50 Hz (20 ms frame rate) for 16,000 frames using MetaMorph software (version 7.10.2, Molecular Devices). Cells were imaged in isotonic buffer (145 mM NaCl, 5 mM KCl, 1.2 mM Na_2HPO_4, 10 mM D-glucose, 20 mM HEPES, pH 7.4, 300 mOsm/L). Time-lapse TIRF movies were captured at 50 Hz (20 ms exposure per frame) at 37°C. To spatially distinguish and temporally separate tracer (**10**), we used 1 nM of the peptide and performed SPT. The power of the laser was sufficient to visualize and track $Cy5_s$ emissions for a short time before they bleached (70% of the initial laser power of the 642 nm laser).

Tracking and diffusion analysis. The single-molecule localization and tracking were extracted from acquisitions acquired at 50 Hz (20 ms) for 16,000 frames (total acquisition time 320 s) using the PALMtracer software[13] as previously described.[14-17] Tracking parameters were set to allow a single detection to be given a maximum displacement of 3 pixels per frame (0.318 µm) to be considered the same detection in the next frame (n+1) for the purpose of tracking. The means squared displacement (MSD, $\mu m^2/s$) and DCoef were calculated from each track with a minimum duration of 8 frames (0.14 s). The diffusion coefficient, DCoef (D, $\mu m^2 s^{-1}$), was calculated from the first 4 points of the MSD. The average number of tracks (per cell) observed in HEK293-hOTR-GFP cells was 4884 ± 1718 (N=9 cells, from 3 independent experiments). Super-resolved images (**Figure 7b**) were generated using the NASTIC software.[18]

FACS. Labeled cells were sorted using the BD FACSAria Fusion flow cytometer (BD Biosciences). To exclude debris, cells were first gated in the forward (FSC-A) *vs*. side (SSC-A) scatter plot. Duplicates were then excluded using the FSC-H vs. FSC-A plot. Subsequently, $Cy3_s$-negative and $Cy3_s$-positive cells were sorted in FACS tubes in purity mode. The purity of the sorted cells was >98%. 5,000 to 10,000 cells were sorted per sample.

RNA extraction and quantitative PCR. After collecting sorted cells in lysis buffer (Bio-Rad), RNA was extracted with an Aurum Total RNA Mini Kit (Bio-Rad). Purified RNA concentrations were measured on a NanoDrop 2000 (Thermo Fisher Scientific) and reverse transcribed to cDNA with the High-Capacity cDNA Reverse Transcription Kit (Applied Biosystems, Thermo Fisher Scientific). Samples were prepared for real-time quantitative PCR (CFX Connect, Bio-Rad) with SYBR Green Master Mix (Bio-Rad) and the following oligonucleotides: *OXTR* forward - TGA AGC ACC TAA GCA TCG CC; *OXTR* reverse - GGT GAT GTC CCA CAG CAA CT (74 bp); *AVPR1A* forward - TGG GCG CCT TTC TTC ATC AT; *AVPR1A* reverse AGG GTT TTC CGA TTC GGT CC (75 bp); and TATA-binding protein (*TBP*) forward - CGG CTG TTT AAC TTC GCT TCC; *TBP* reverse - GAG CAT CTC CAG CAC ACT CTT (129 bp) as housekeeping gene. Samples were loaded as triplicates, and relative gene expression ($\Delta\Delta$Ct) was calculated with the CFX Maestro software (BioRad) and normalized to the lowest value. Finally, qPCR samples were loaded on a 1.5% agarose gel to verify the correct product size (see **Figure S11** for full blot).

Primary UtSMC and hippocampal neuronal cultures. UtSMC cells were a kind gift from Prof. Sandra Hayder (Department of Obstetrics and Gynecology, Medical University of Vienna, Austria) and maintained in Smooth Muscle Cell Basal Medium 2 (PromoCell), including supplements (PromoCell) and P/S (Gibco) at 37°C and 5% CO_2. Cells were trypsinized with 0.1% Trypsin/EDTA (Gibco) and plated on glass-bottom 96-well plates (CellVis) at a density of 10,000 cells/well in full growth medium. The next day, cells were stimulated with tracer (**11**), OTA, or scrambled peptide and either imaged in an IncuCyte SX5 system for 45 min or fixed and counterstained with Hoechst 33,342 after 60 min of treatment for confocal microscopy on a Zeiss LSM880 confocal microscope.

For neuronal cultures, hippocampi were dissected from fetal E16.5 mice and E17.5 rat brains and mechanically dissociated with flame-polished Pasteur pipettes in Hank's balanced salt solution (HBSS, Gibco). Tissue pieces were subsequently enzymatically dissociated with 0.1% Trypsin/EDTA (Gibco) in Neurobasal A (NBA, Gibco) containing 1,000U/mL DNase (Sigma) for 5 min at 37°C. Cells were triturated with flamed Pasteur pipettes, filtered through a 40 µm nylon mesh (Thermo Fisher Scientific), centrifuged at 200 *g* for 5 min, and washed up to three times in NBA containing P/S (Gibco), GlutaMax (Gibco), and B27 supplement (Gibco). Cells were plated at 20,000 cells/well on PDL-coated (Sigma) glass-bottom black 96-well microscopy plates (Cellvis) and grown at 37°C with 5% CO_2. Half of the medium was replaced every 3-4 days. After 13-14 days *in vitro*, neuronal cultures were exposed to 100 nM tracer (**11**) for one hour, with or without 30-min pretreatment with 10 µM of the peptide antagonist OTA. In parallel, neurons were exposed to 100 nM of the scrambled tracer SCR for 1 h. After stimulations, cells were washed with ice-cold PBS and fixed with 4% paraformaldehyde for 30 min on ice. Next, cells were washed with PBS, counterstained with the nuclear marker Hoechst 33,342 (1:5,000 in PBS for 10 min), washed again in PBS, and directly imaged on a Zeiss LSM880 confocal microscope.

II. Supporting Tables

Table S1. Compound characterization summary.

	Conc.	PC	Yield	Purity	Ret. time	Calc. [m/z]	Obs. [m/z]
**OT** CYIQNCPLG*	1871 µM	63%	50%	97.5%	16.6 min	$[M+2H]^{2+}$ 504.2255	$[M+2H]^{2+}$ 504.2257
**VP** CYFQNCPRG*	1884 µM	68%	47%	>99.0%	14.8 min	$[M+2H]^{2+}$ 542.7262	$[M+2H]^{2+}$ 542.7263
**OTA** $d(CH_2)_5$yITNCP(Orn)	2178 µM	71%	14%	97.3%	20.2 min	$[M+H]^{+}$ 978.4423	$[M+H]^{+}$ 978.4424
**SCR** dCINYQCP(Orn)G*-[d-PEG_5-k^{ε}-$Cy3_s$]^8	765 µM	55%	8%	>99.0%	17.5 min	$[M+2H]^{2+}$ 1070.9624	$[M+2H]^{2+}$ 1070.9617
**1** d(Orn)^{8}OT	1786 µM	59%	46%	96.3%	15.6 min	$[M+H]^{+}$ 993.4281	$[M+H]^{+}$ 993.4284
**2** d(Orn)^{8}OT-[$Cy5_s$]^8	709 µM	39%	24%	97.0%	18.4 min	$[M+2H]^{2+}$ 816.3237	$[M+2H]^{2+}$ 816.3239
**3** d(Orn)^{8}OT-[PEG_5-$Cy5_s$]^8	810 µM	52%	10%	95.2%	18.7 min	$[M+2H]^{2+}$ 962.4093	$[M+2H]^{2+}$ 962.4091
**4** d(Orn)^{8}OT-[PEG_5-$Cy3_s$]^8	773 µM	49%	15%	97.0%	17.8 min	$[M+2H]^{2+}$ 949.4014	$[M+2H]^{2+}$ 949.4022
**5** d(Orn)^{8}OT-[k^{ε}-k^{ε}-$Cy5_s$]^8	635 µM	40%	2%	97.3%	17.9 min	$[M+2H]^{2+}$ 944.9201	$[M+2H]^{2+}$ 944.9200
**6** d(Orn)^{8}OT-[k^{ε}-k^{ε}-$Cy3_s$]^8	655 µM	41%	2%	97.9%	16.9 min	$[M+2H]^{2+}$ 931.9123	$[M+2H]^{2+}$ 931.9120
**7** d(Orn)^{8}OT-[PEG_5-k^{ε}-$Cy5_s$]^8	389 µM	27%	9%	96.9%	18.6 min	$[M+2H]^{2+}$ 1026.4568	$[M+2H]^{2+}$ 1026.4564
**8** d(Orn)^{8}OT-[PEG_5-k^{ε}-$Cy3_s$]^8	625 µM	42%	22%	96.7%	17.7 min	$[M+2H]^{2+}$ 1013.4489	$[M+2H]^{2+}$ 1013.4495
**9** d(Orn)^{8}OT-[PEG_5-PEG_5-k^{ε}-$Cy3_s$]^8	357 µM	28%	6%	97.4%	18.0 min	$[M+2H]^{2+}$ 773.0244	$[M+2H]^{2+}$ 773.0246
**10** d(Orn)^{8}OT-[d-PEG_5-$Cy5_s$]^8	673 µM	46%	7%	95.2%	18.6 min	$[M+2H]^{2+}$ 1019.9226	$[M+2H]^{2+}$ 1019.9227
**11** d(Orn)^{8}OT-[d-PEG_5-k^{ε}-$Cy3_s$]^8	800 µM	57%	4%	>99.0%	17.6 min	$[M+2H]^{2+}$ 1070.9624	$[M+2H]^{2+}$ 1070.9615
**12** d(Orn)^{8}OT-[d-PEG_5-k^{ε}-AF488]^8	988 µM	67%	4%	>99.0%	15.3 min	$[M+2H]^{2+}$ 1022.8609	$[M+2H]^{2+}$ 1022.8604
**13** d(Orn)^{8}OT-[d-PEG_5-k^{ε}-TAMRA]^8	1358 µM	88%	5%	98.6%	19.7 min	$[M+3H]^{3+}$ 647.6260	$[M+3H]^{3+}$ 647.6279

Abbreviations: $Cy3_s$ = sulfo-cyanine 3; $Cy5_s$ = sulfo-cyanine 5; 'd' if at the beginning of sequence = desamino (no N-terminal amine); 'd' if at any other part of the sequence = D-aspartic acid; $d(CH_2)_5$ = 1-[*β*-mercapto-*β,β*-cyclopentamethylene]propionic acid; k^{ε} = next building block attached to ε-amine; Orn = ornithine; OT = oxytocin; OTA = oxytocin receptor antagonist; PC = peptide content; PEG_5 = polyethylene glycol 5; SCR = $Cy3_s$-labeled scrambled peptide; VP = vasopressin; * = C-terminal amide.

III. Supporting Figures

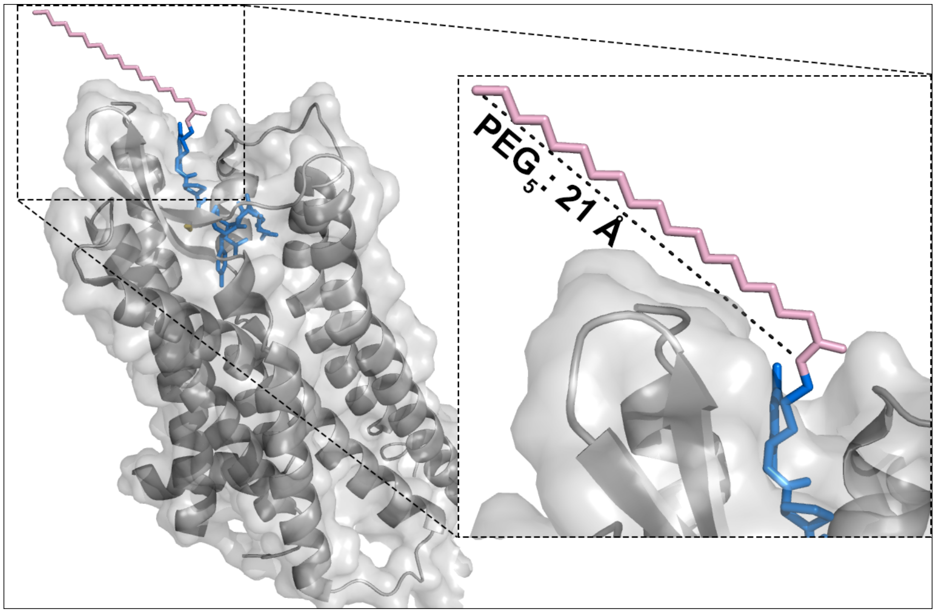

Figure S1. Estimation of required linker length for d(Orn)^{8}OT-based tracers using PyMOL. Attachment of PEG_5 to the ornithine side chain resulted in a 21 Å distance between the parent peptide and bulky fluorophores in the stretched conformation, which was considered sufficient for minimizing interactions between the receptor and fluorophores.

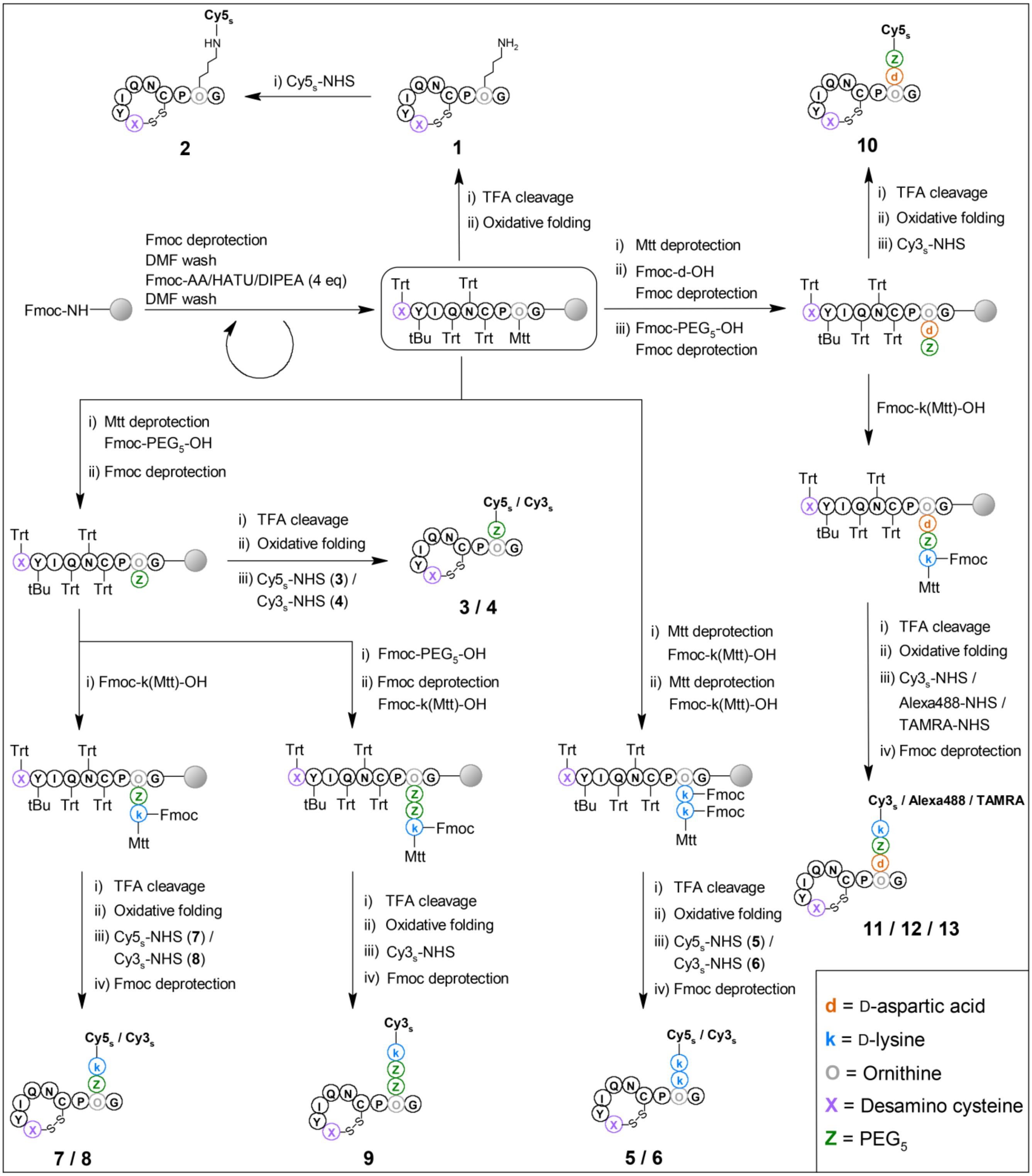

Figure S2. Synthetic strategy to produce the different fluorescent OTR tracers. Peptide and linker assemblies were carried out *via* Fmoc-SPPS, followed by TFA global sidechain deprotection and resin cleavage, oxidative folding, and in-solution fluorophore attachment using NHS-activated dyes. Linker building blocks ornithine (O, gray), D-lysine (k, blue), PEG_5 (Z, green), and D-aspartic acid (d, orange) were colored accordingly.

OT: H_2N-CYIQNCPLG-$CONH_2$

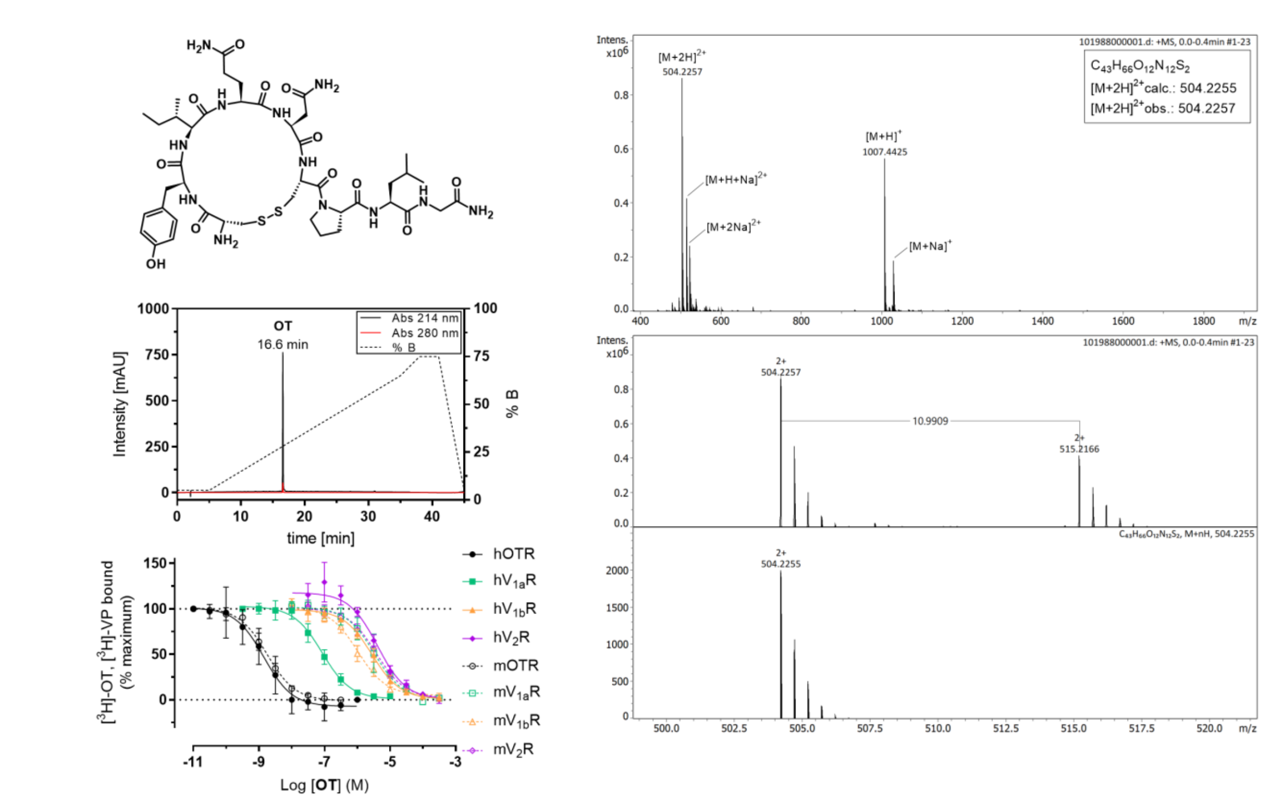

VP: H_2N-CYFQNCPRG-$CONH_2$

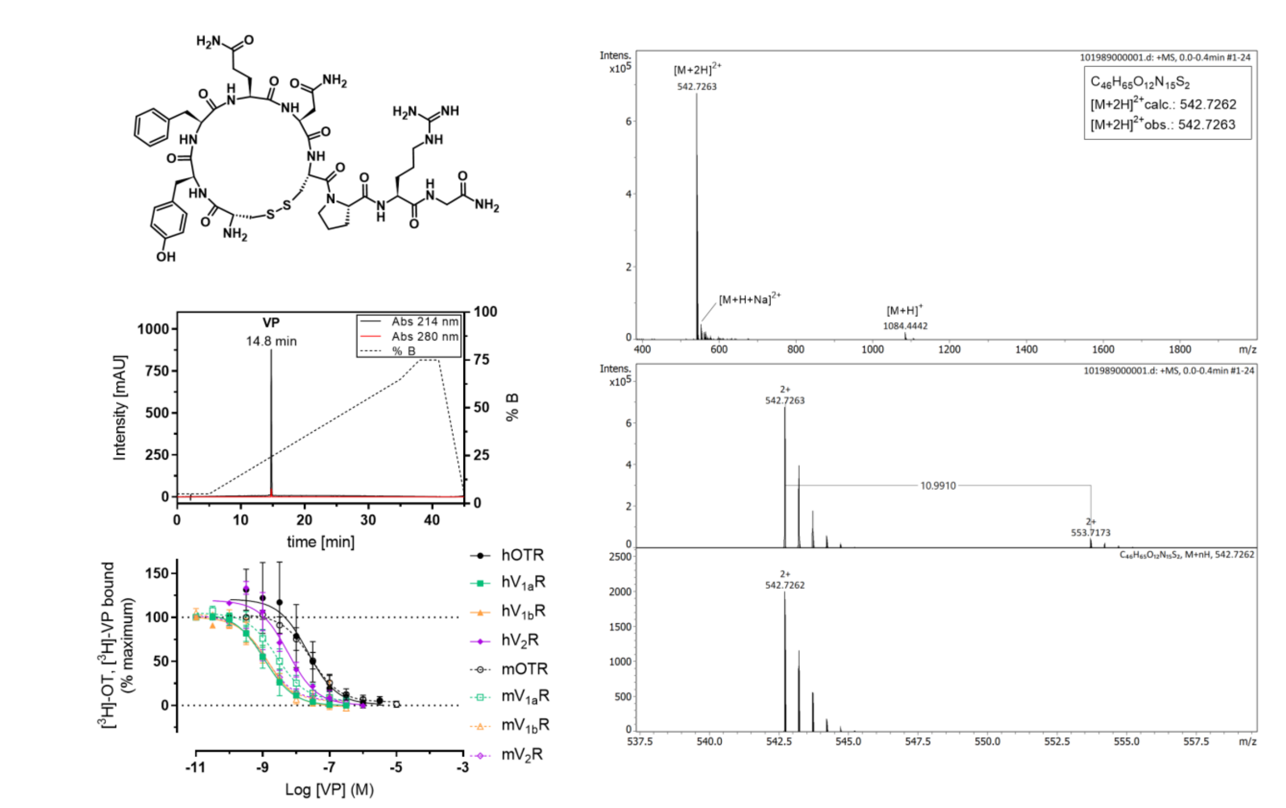

OTA: d(CH_2)$_5$yITNCP(Orn)-COOH

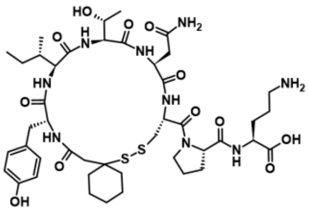

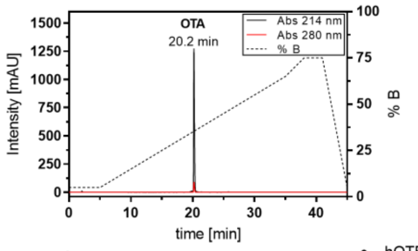

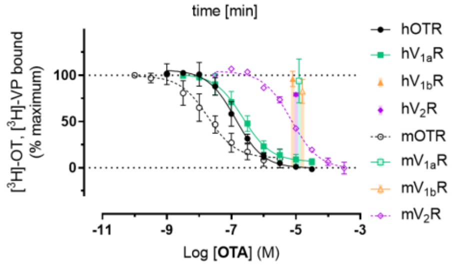

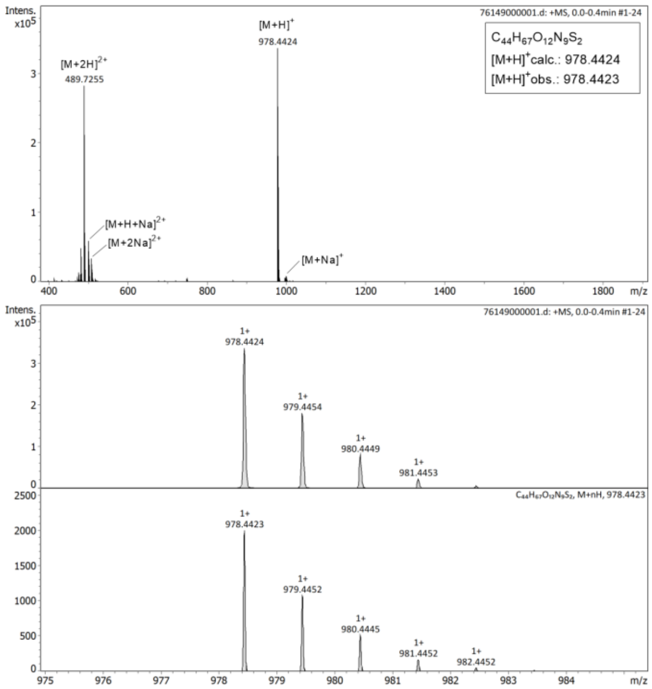

SCR: dClNYQCP(Orn$^{\delta}$-d-PEG$_5$-k$^{\varepsilon}$-Cy3$_s$)G-$CONH_2$

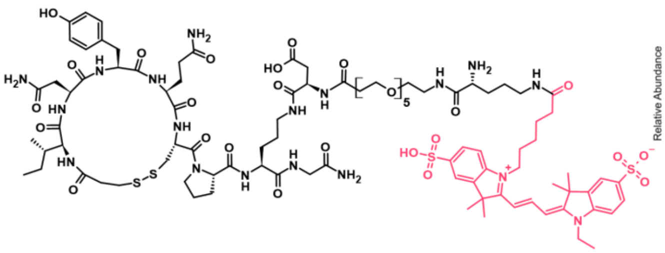

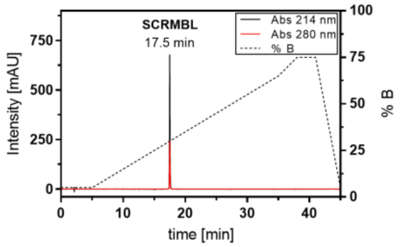

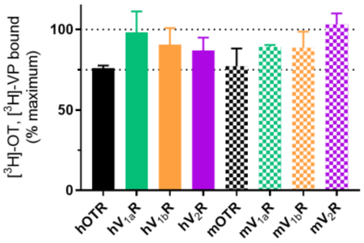

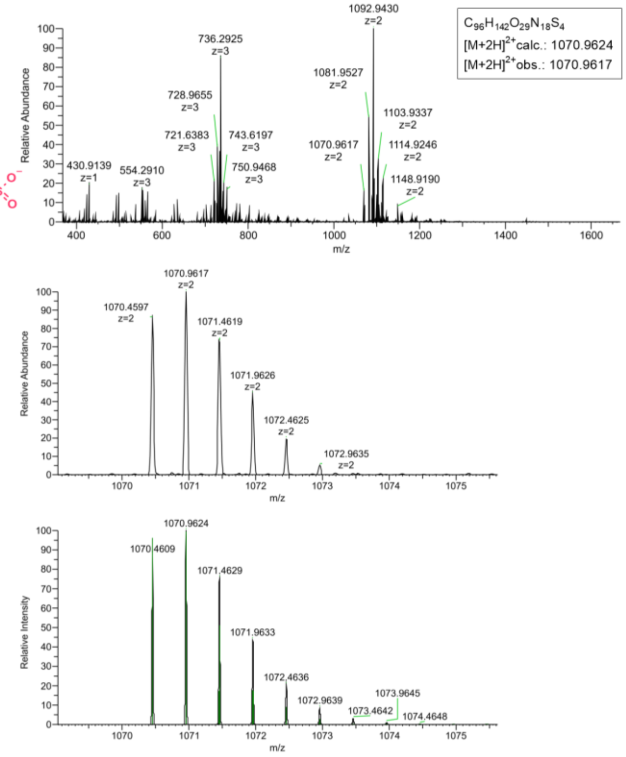

1: dCYIQNCP(Orn)G-CONH$_2$

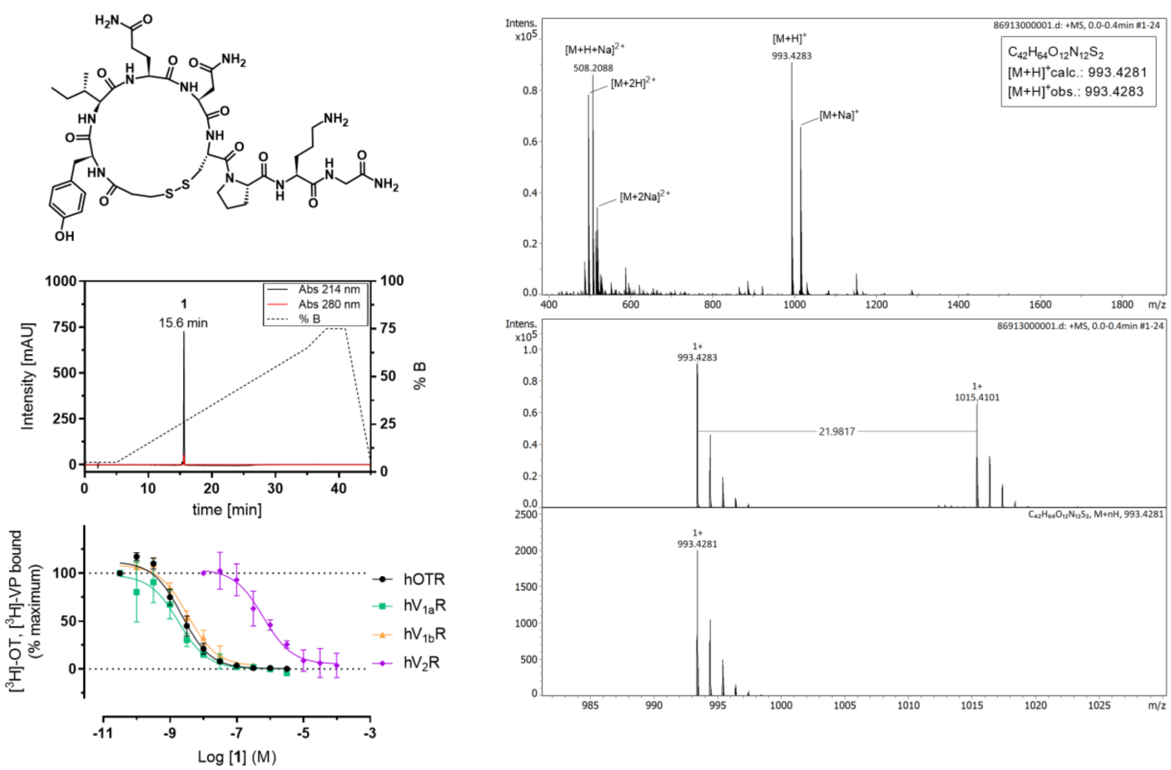

2: dCYIQNCP(Orn$^{\delta}$-Cy5s)G-CONH$_2$

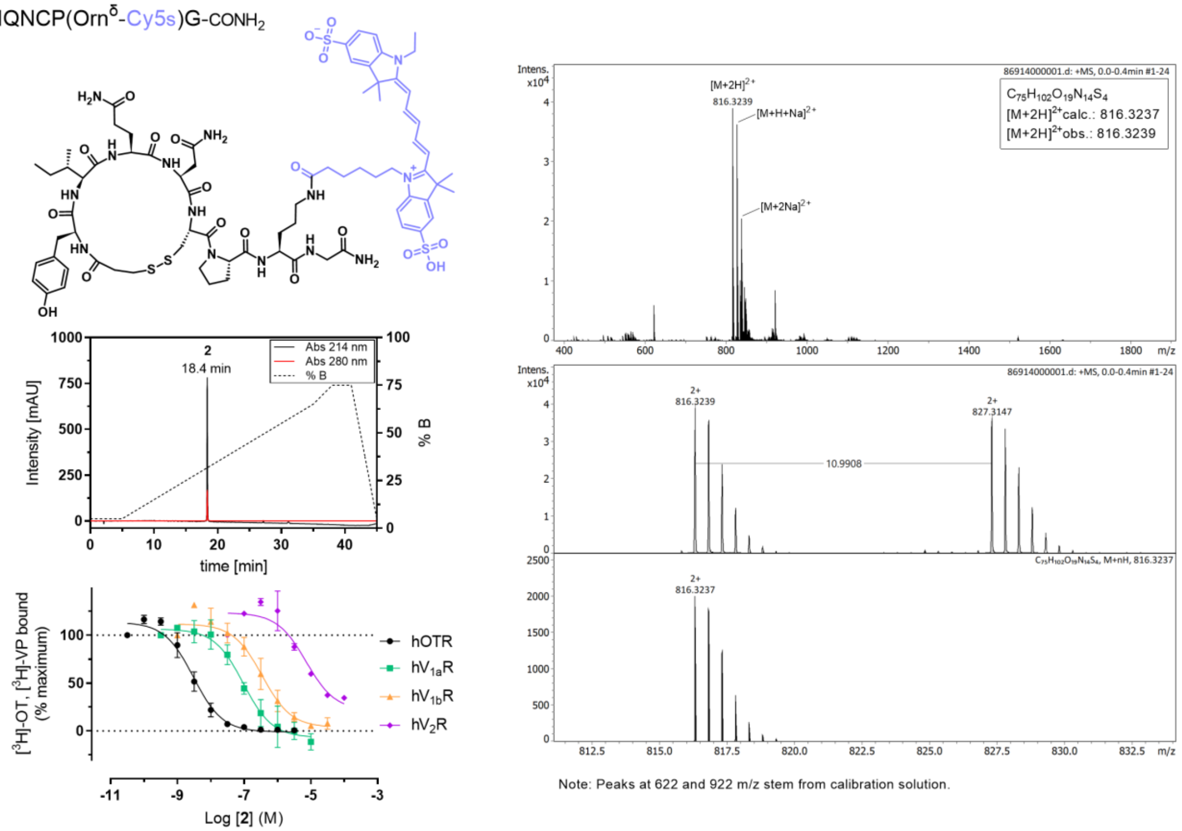

Note: Peaks at 622 and 922 m/z stem from calibration solution.

3: dCYIQNCP(Orn$^{\delta}$-PEG$_5$-Cy5s)G-CONH$_2$

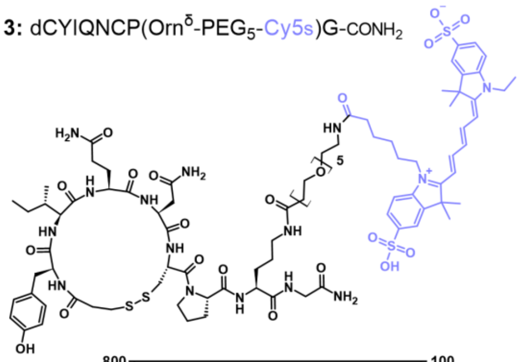

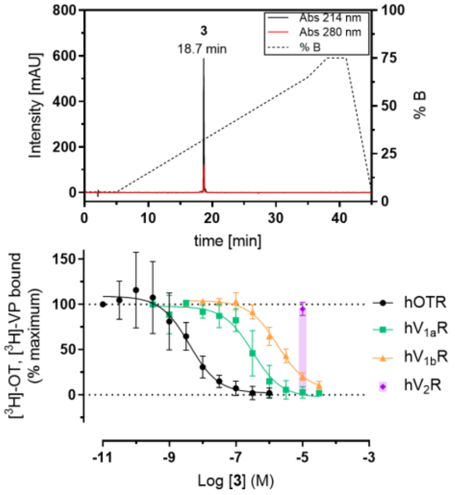

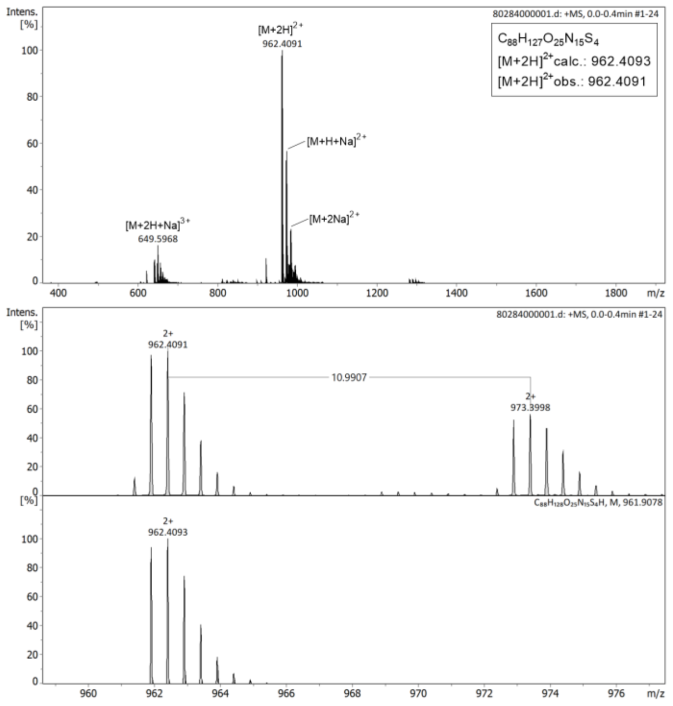

Note: Peaks at 622 and 922 m/z stem from calibration solution.

4: dCYIQNCP(Orn$^{\delta}$-PEG$_5$-Cy3$_s$)G-CONH$_2$

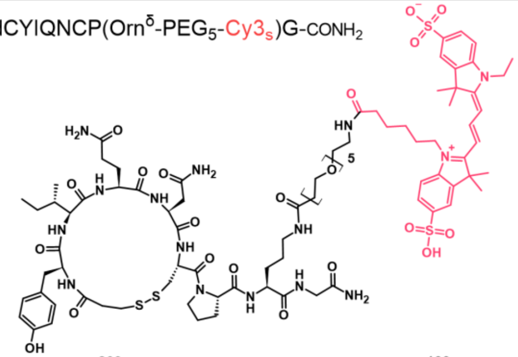

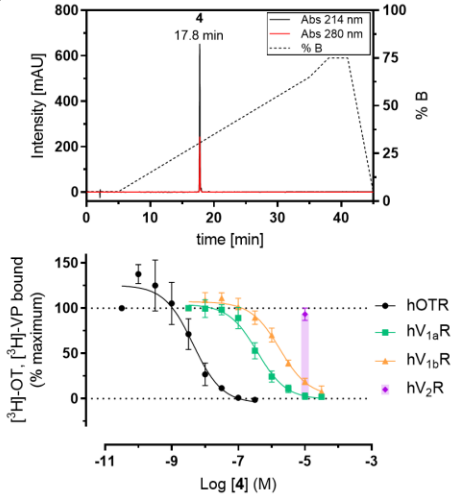

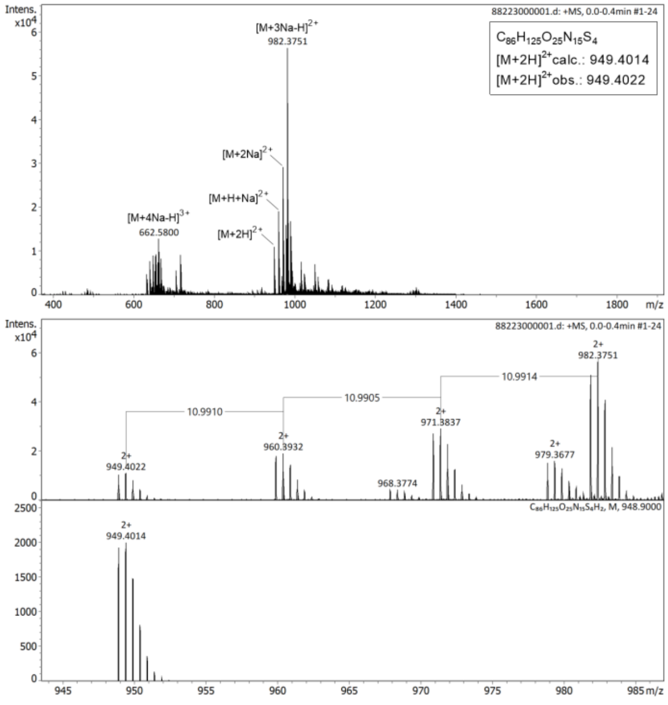

5: dCYIQNCP(Orn$^{\delta}$-k$^{\varepsilon}$-k$^{\varepsilon}$-Cy5$_s$)G-CONH$_2$

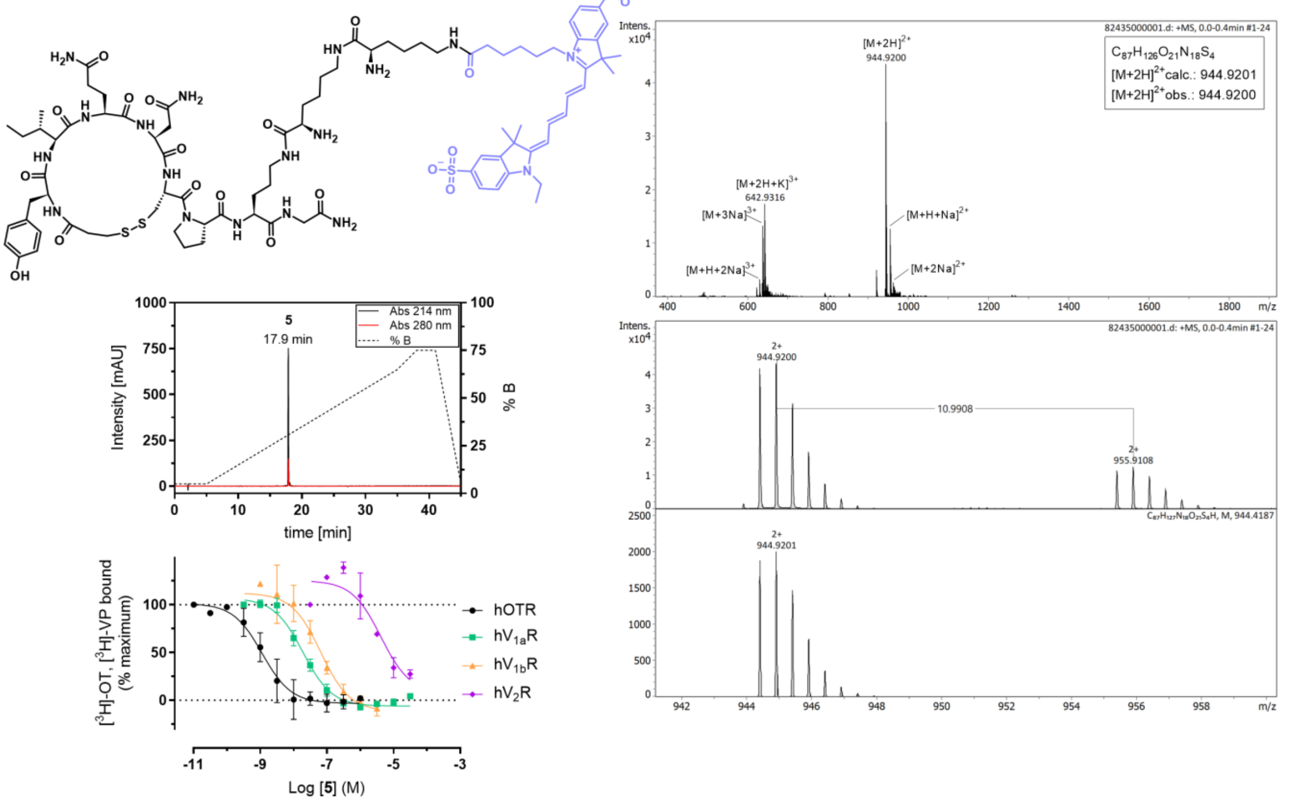

6: dCYIQNCP(Orn$^{\delta}$-k$^{\varepsilon}$-k$^{\varepsilon}$-Cy3$_s$)G-CONH$_2$

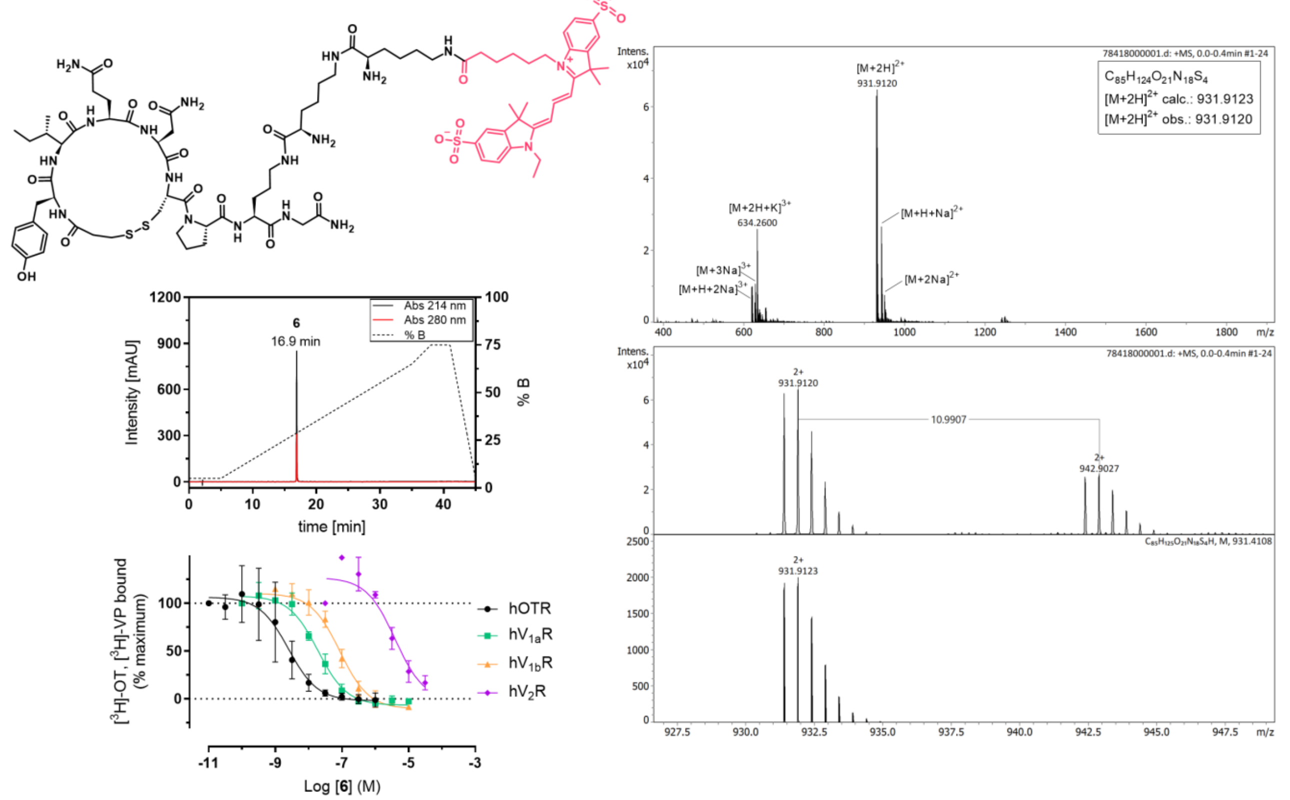

7: dCYIQNCP(Orn$^{\delta}$-PEG$_5$-k$^{\varepsilon}$-Cy5$_s$)G-CONH$_2$

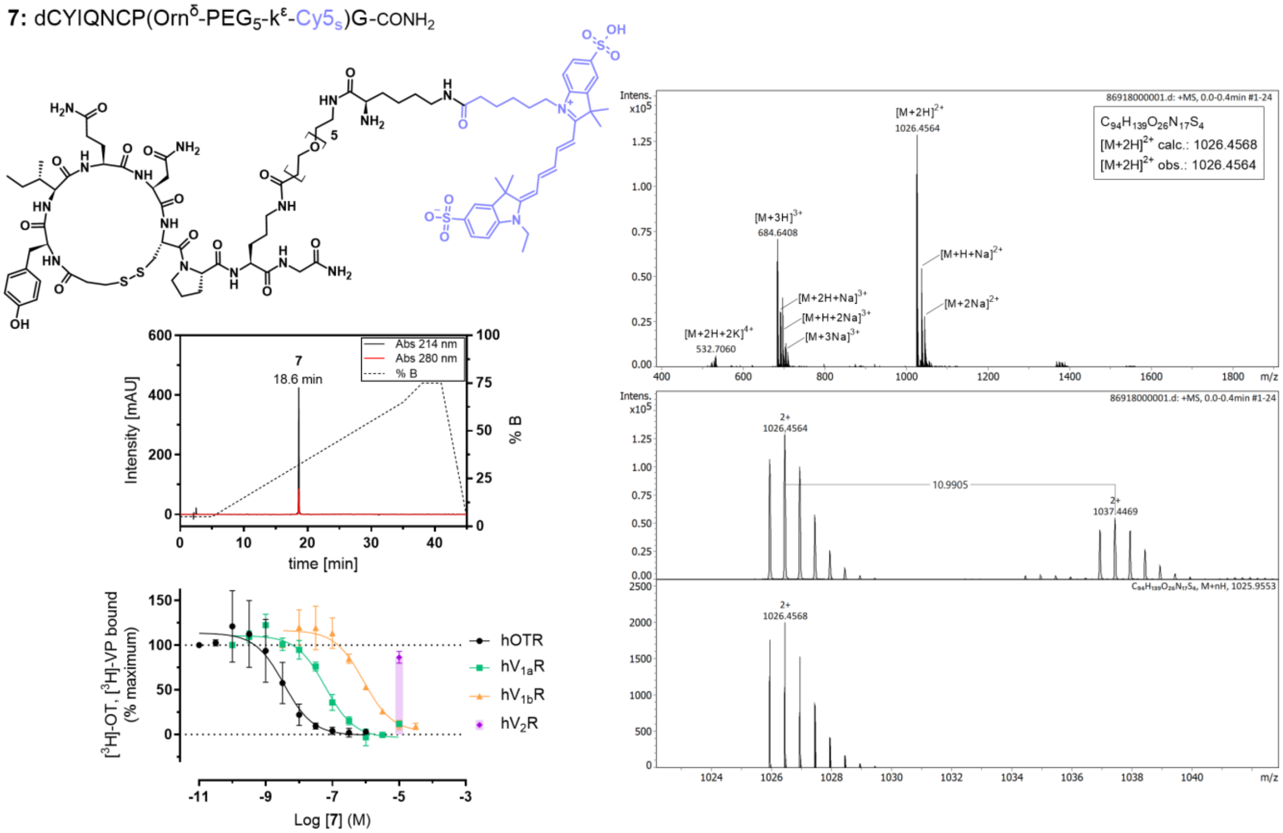

8: dCYIQNCP(Orn$^{\delta}$-PEG$_5$-k$^{\varepsilon}$-Cy3$_s$)G-CONH$_2$

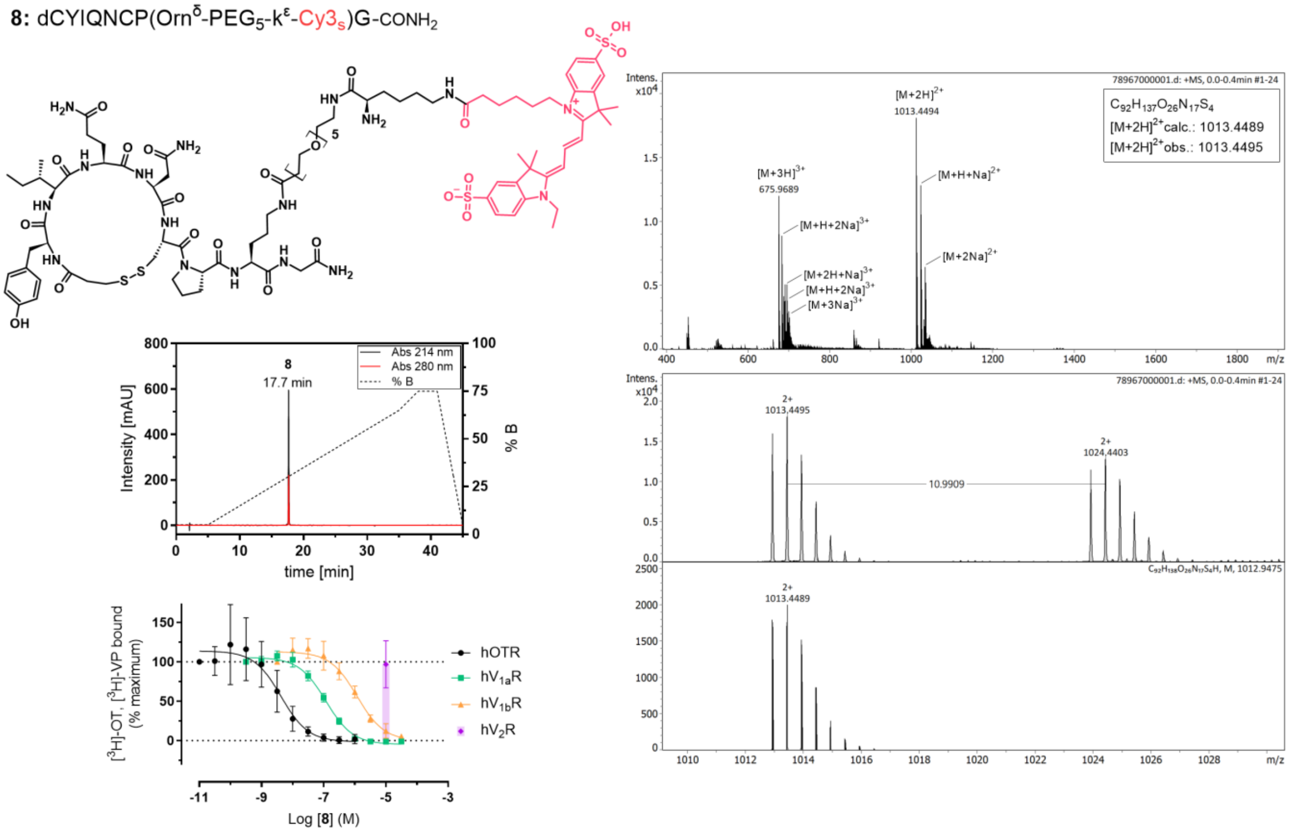

9: dCYIQNCP(Orn$^{\delta}$-PEG$_5$-PEG$_5$-k$^{\varepsilon}$-Cy3$_s$)G-CONH$_2$

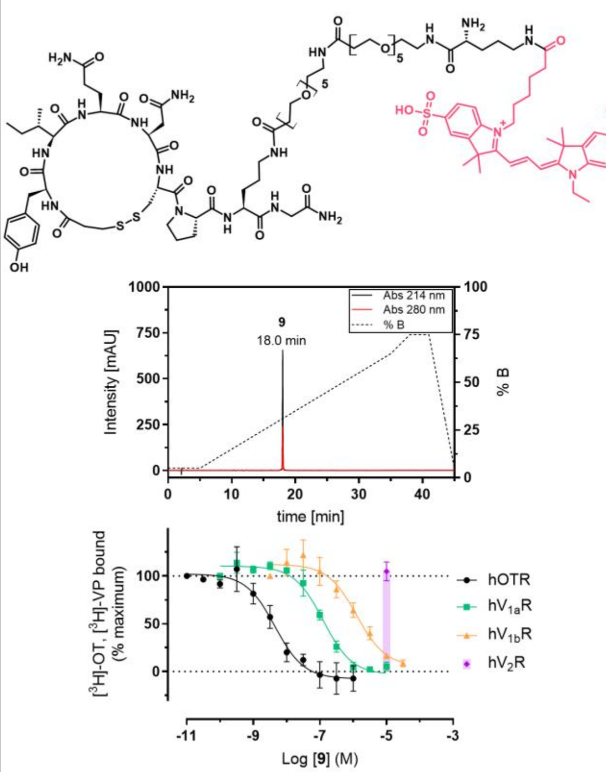

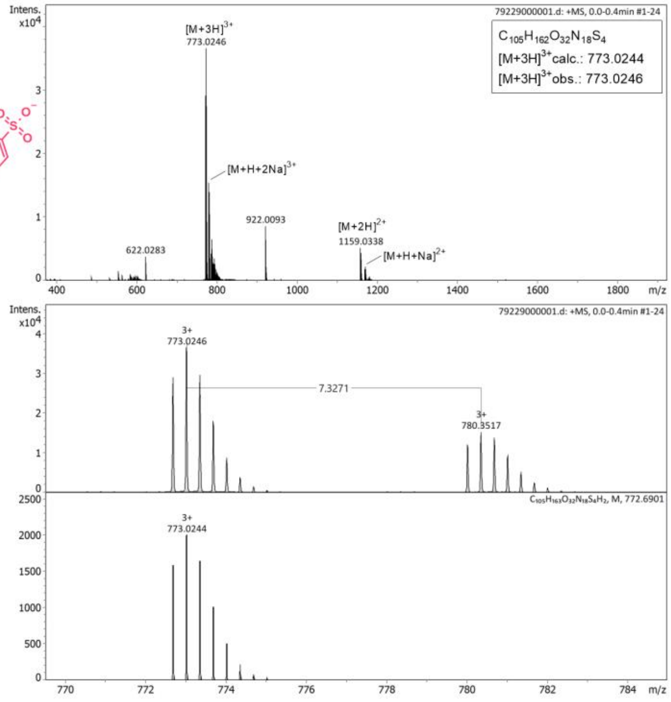

Note: Peaks at 922 and 622 m/z stem from calibration solution.

10: dCYIQNCP(Orn$^{\delta}$-d-PEG$_5$-Cy5$_s$)G-CONH$_2$

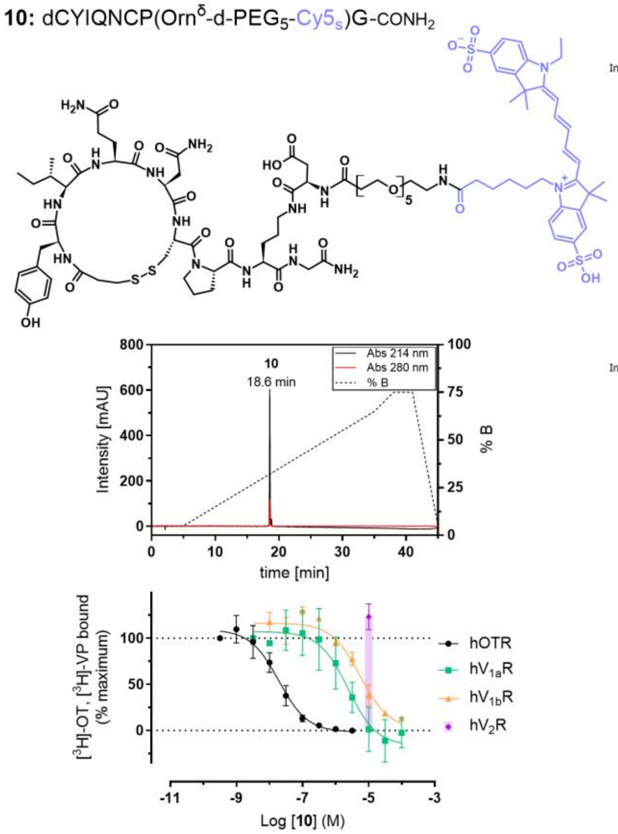

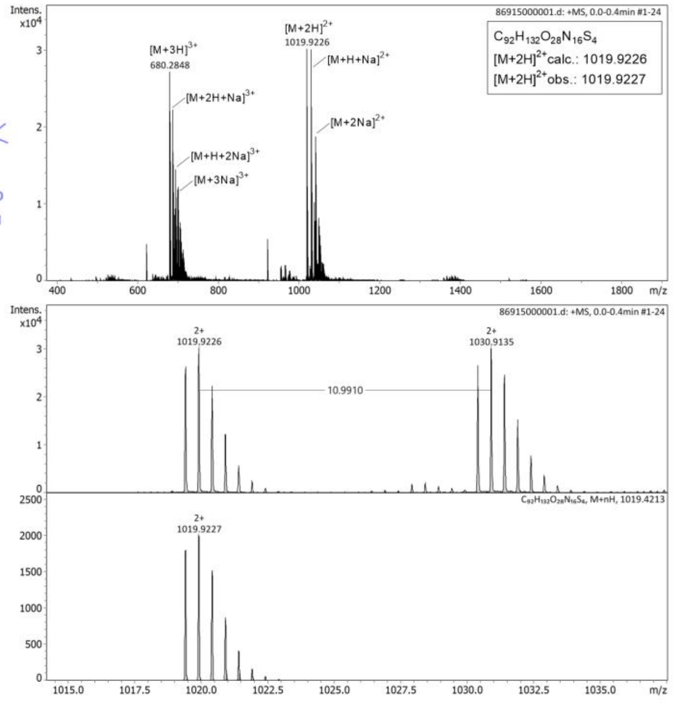

Note: Peaks at 922 and 622 m/z stem from calibration solution.

11: dCYIQNCP(Orn$^{\delta}$-d-PEG$_5$-k$^{\varepsilon}$-Cy3$_s$)G-CONH$_2$

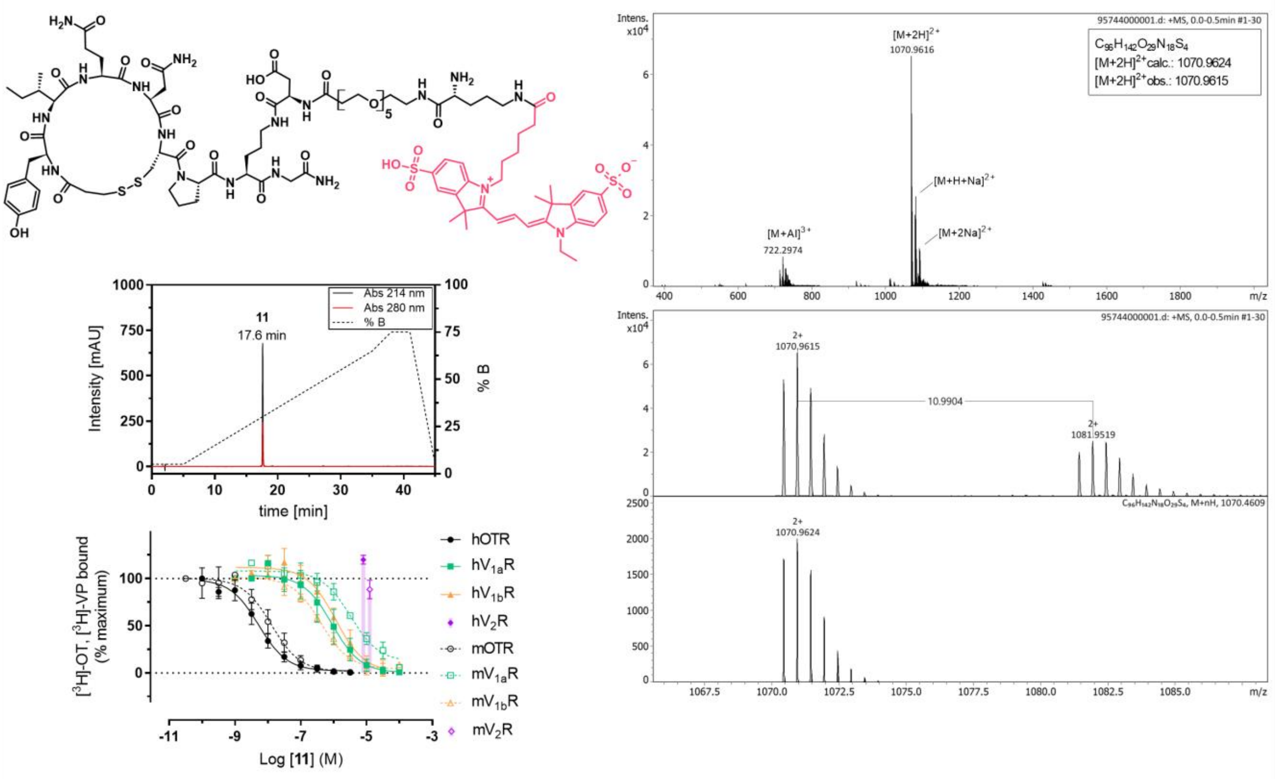

12: dCYIQNCP(Orn$^{\delta}$-d-PEG$_5$-AF488)G-CONH$_2$

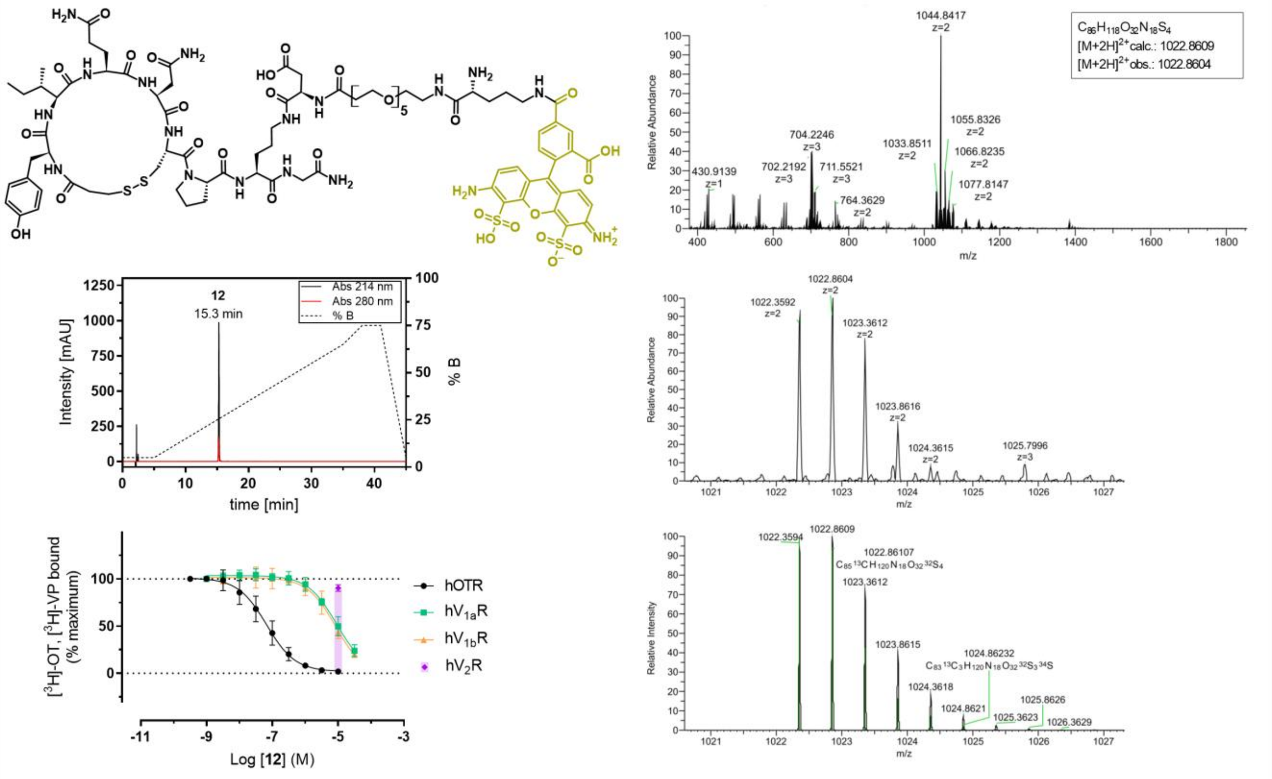

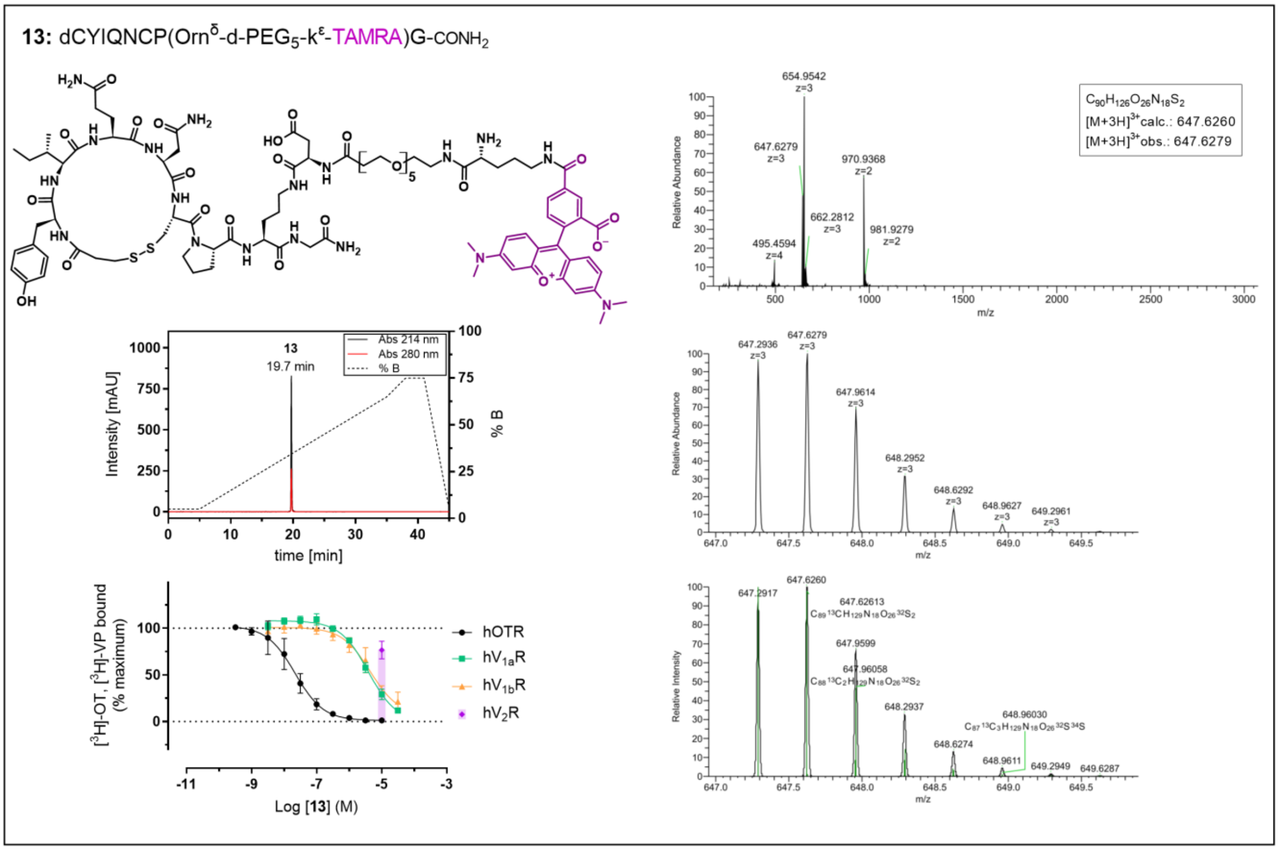

Figure S3. Overview of compound structures, purity, radioligand displacement curves, and high-resolution mass spectra. Analytic C_{18}-RP-HPLC chromatograms were used for retention time determination and product purity evaluation at 214 nm. Solvent A (ddH$_2$O + 0.1% TFA) and B (ACN + 0.08% TFA) were used as eluents at 1 mL/min flow rate and a linear gradient of 5-65% B in 30 min. All final products had a purity of >95%. Dose-response curves were obtained from radioligand displacement experiments of membrane preparations of HEK293 cell lines applying [^{3}H]-OT at OTR and [^{3}H]-VP at V_{1a}R, V_{1b}R, and V_2R. Values were normalized to the percentage of maximum binding (i.e., no competing ligand present). Specific binding was obtained from the subtraction of non-specific binding from total binding. K_i values were derived from one-site-fitted competition binding curves. For compounds that did not displace the radioligand to more than 25% at concentrations of 10 µM (shown as one-point binding) the K_i was denoted as >10 µM. Data are presented as mean ± SD from at least three independent experiments for dose-response curves and two independent experiments for one-point displacements. Final products were confirmed through HR-ESI-MS direct injections on a maXis HD ESI-Qq-TOF mass spectrometer.

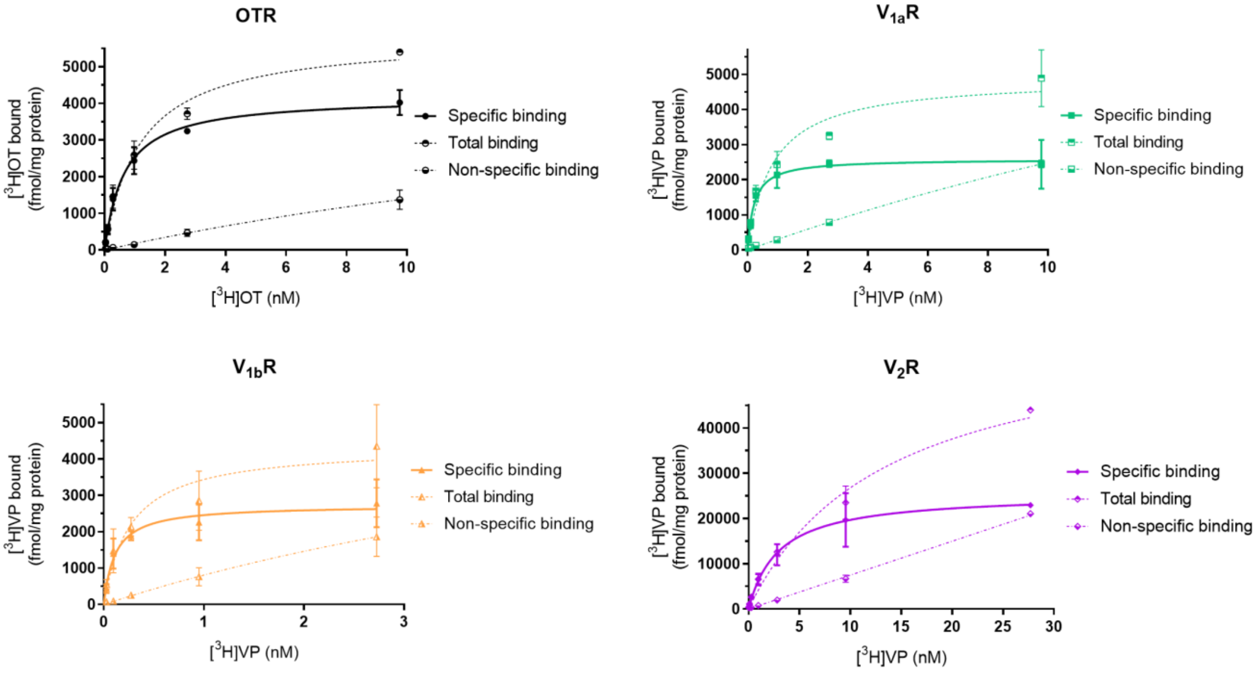

Figure S4. Saturation bindings of [^{3}H]-OT and [^{3}H]-VP using the stable human OTR-GFP, V_{1a}R-GFP, V_{1b}R-GFP, and V_2R-GFP HEK293 cell lines. Distinct concentrations of radioligands were incubated for 1 h at 37°C with membrane preparations of the respective stable cell lines in the presence of saturating concentrations of competing ligands (10 µM of OT or VP) in accordance with published procedures.[19] K_d and B_{max} values were derived from one site-fitted saturation binding curve and were 0.65 nM and 4.2 pmol/mg for [^{3}H]OT at hOTR, 0.21 nM and 2.6 pmol/mg for [^{3}H]VP at hV_{1a}R, 0.14 nM and 2.9 pmol/mg for [^{3}H]VP at hV_{1b}R, and 2.95 nM and 25.5 pmol/mg for [^{3}H]VP at hV_2R respectively. Data are presented as mean ± SD from at least two independent experiments.

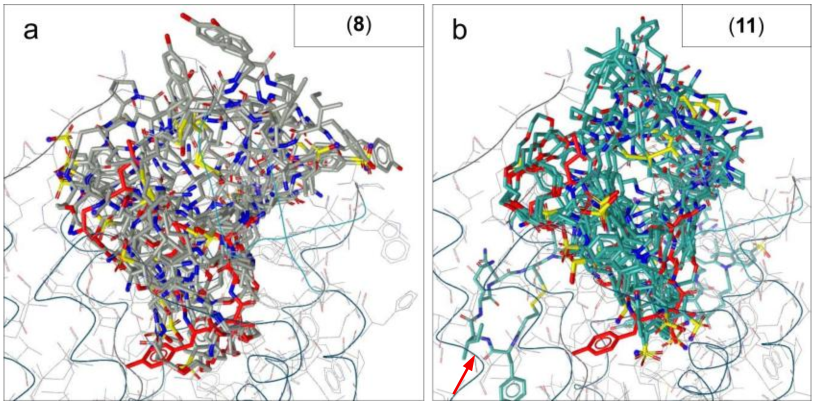

Figure S5. Tracer docking study to human OTR. (**a,b**) Docking of compounds d(Orn)^{8}OT-[PEG$_5$-k^ε-Cy3$_s$]^8 (**8**) and d(Orn)^{8}OT-[d-PEG$_5$-k^ε-Cy3$_s$]^8 (**11**) did not result in configurations in which the macrocycles were located in the binding pocket in a comparable orientation as the original macrocycle of d(Orn)^{8}OT or OT. Although docking of these compounds resulted in low energy structures with comparable binding affinity estimations, most of the time, the linker as well as the fluorophores are positioned within the binding site, and the macrocycle interacts with the receptor amino acids outside of the binding pocket (*red arrow*, **b**). The original position of OT is shown in red for comparison.

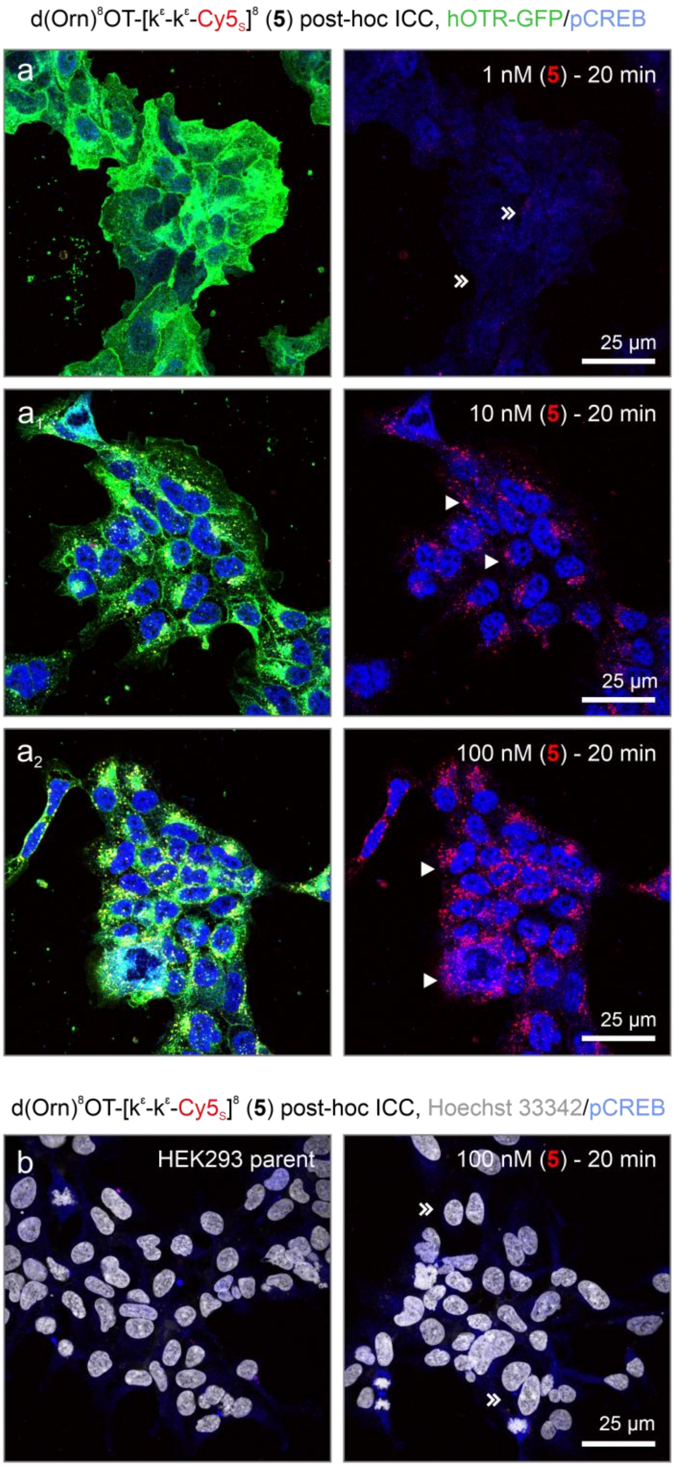

Figure S6. Imaging capacity of d(Orn)^{8}OT-[k$^{\epsilon}$-k$^{\epsilon}$-Cy5$_{s}$]^{8} (5). (**a-a$_2$**) Tracer (**5**) (*red*) dose-dependently induced hOTR-GFP (*green*) internalization and CREB phosphorylation (*blue*). Effects were observed from 10 nM onwards. (**b**) Compound (**5**) at 100 nM did not affect pCREB induction in parent HEK293 cells lacking hOTR, nor was it internalized into the cytoplasm. Nuclei were labeled in *white* (**b**). *Arrowheads* and *double arrowheads* denote pCREB activation or non-response, respectively.

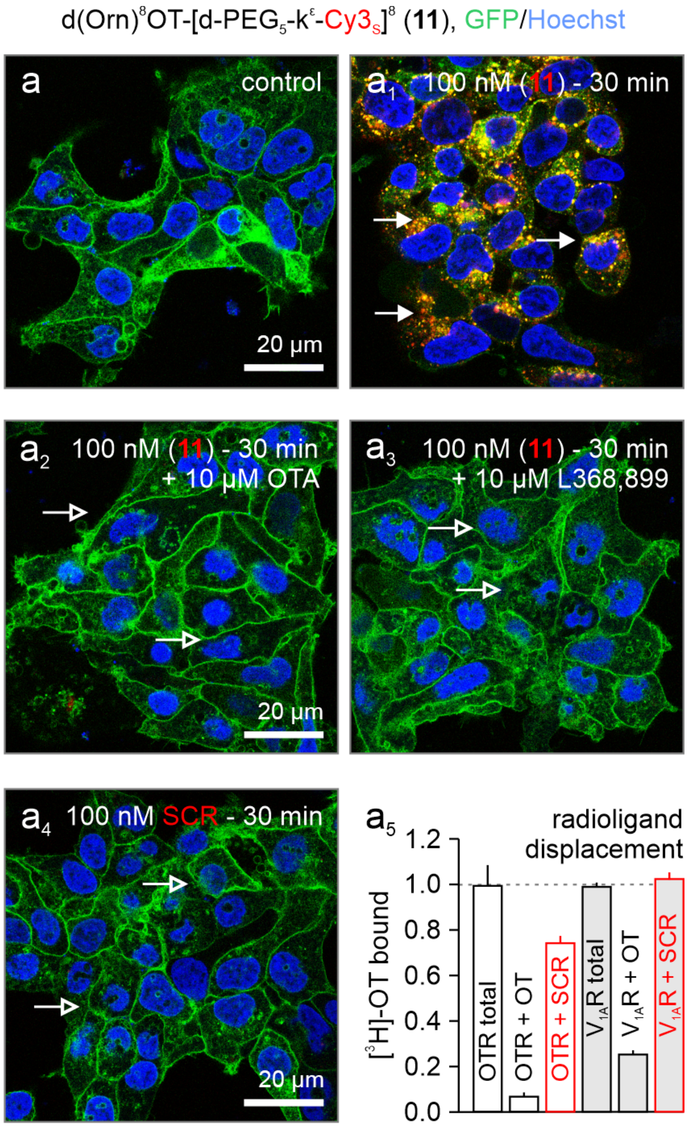

Figure S7. Peptide antagonist OTA, small molecule antagonist L368,899 and labeled scrambled peptide SCR validation in hOTR-GFP cells. (**a-a$_3$**) Pretreatment with 10 µM OTA, as well as 10 µM L368,899 (30 min)[4], completely inhibited the uptake of compound (**11**). (**a$_4$,a$_5$**) SCR (dCINYQCP(Orn)G-[d-PEG$_5$-k$^\varepsilon$-Cy3$_S$]^8) was not taken up by hOTR-GFP cells (100 nM; **a$_4$**), nor displaced 1 nM [^{3}H]-OT binding on OTR, V$_{1a}$R (10 µM, mean ± SD from triplicates; **a$_5$**), V$_{1b}$R and V$_2$R (*main manuscript Table 1*), indicating that the parent peptide is the source of OTR affinity.

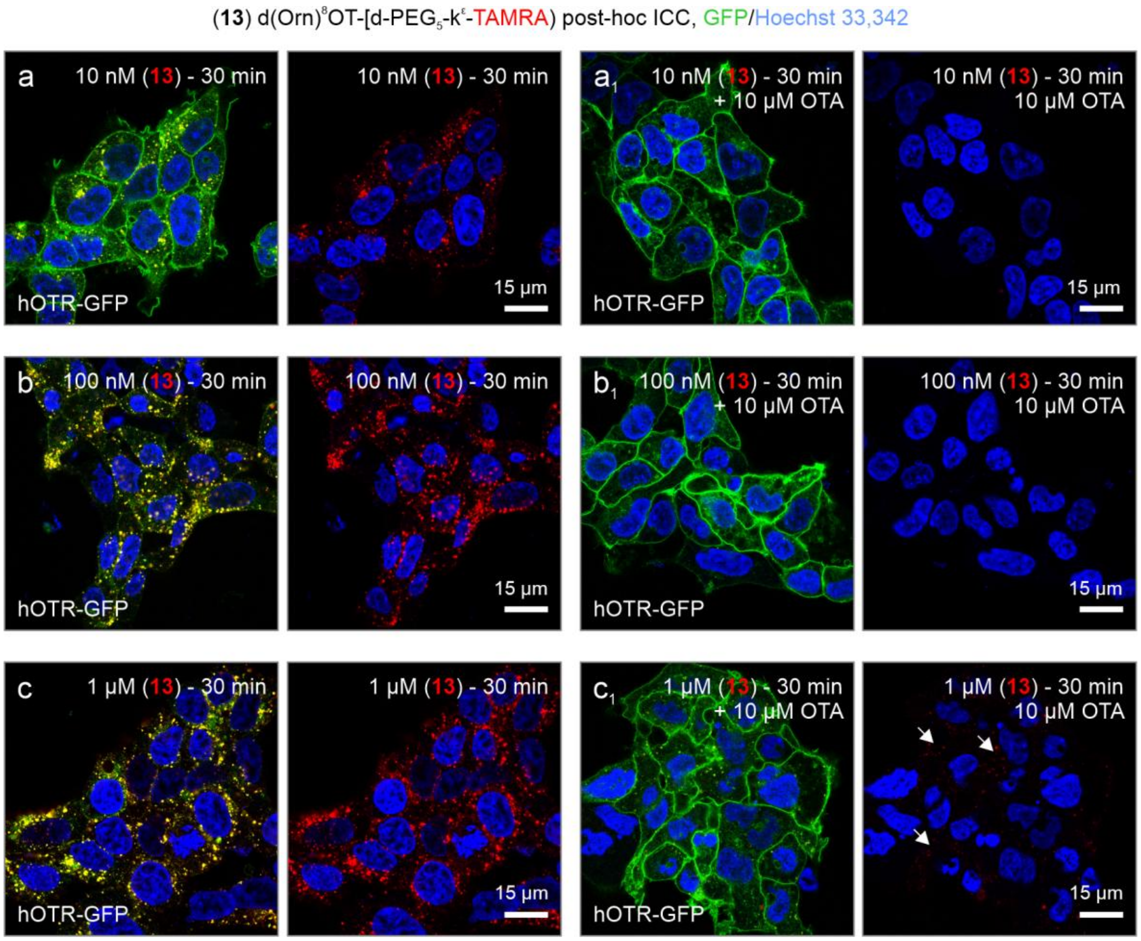

Figure S8. Validation of d(Orn)^{8}**OT-[d-PEG**$_{5}$**-k**$^{\varepsilon}$**-TAMRA]**^{8} **(13).** (**a-c**$_{1}$) Stimulation of HEK293 cells overexpressing hOTR-GFP with tracer (**13**) d(Orn)^{8}OT-[PEG$_{5}$-k$^{\varepsilon}$-TAMRA]^{8} reveals a dose-dependent GFP-internalization and TAMRA accumulation. Pretreatment with 10 µM of the peptide antagonist OTA completely prevented internalization of tracer (**13**) and GFP signals up to 100 nM (**a**$_{1}$,**b**$_{1}$), while at 1 µM, minor uptake was visible (**c**$_{1}$, *arrows*).

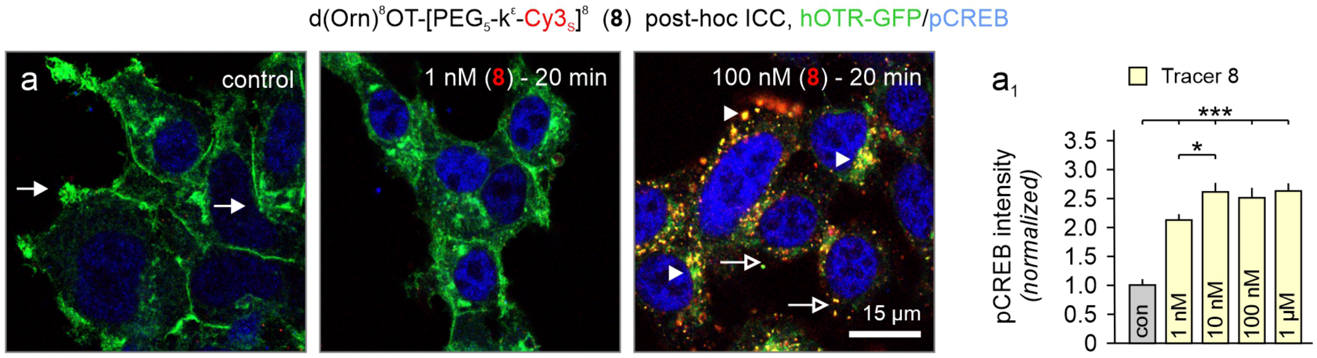

Figure S9. Biological activity of tracer (8) on OTR-expressing cells. (**a**,**a**$_{1}$) Treatment with tracer d(Orn)^{8}OT-[PEG$_{5}$-k$^{\varepsilon}$-Cy3$_{s}$]^{8} (**8**) confirmed a dose-dependent GFP (*closed vs. open arrows*) and compound internalization (*arrowheads*). CREB phosphorylation events were observed from 1 nM onward. Data are presented as MEAN ± SEM (n = ~150 cells over two coverslips) and statistical analysis was done applying Student's t-test (*P<0.05, ***P<0.001).

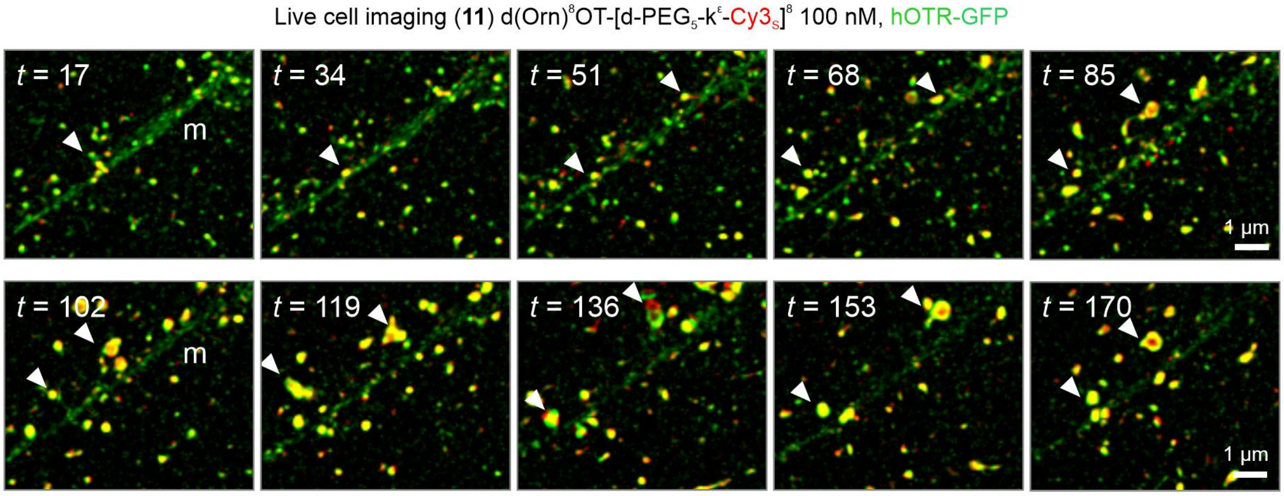

Figure S10. hOTR-GFP receptor trafficking over time in HEK293 cells. The addition of 100 nM tracer (**11**) induced immediate GFP (*green*) and (**11**) (*red*) internalization events (*arrowheads*). Note the removal of GFP signals from the membrane (*m*) after time (*seconds*) and the accumulation of (**11**) in the vesicles.

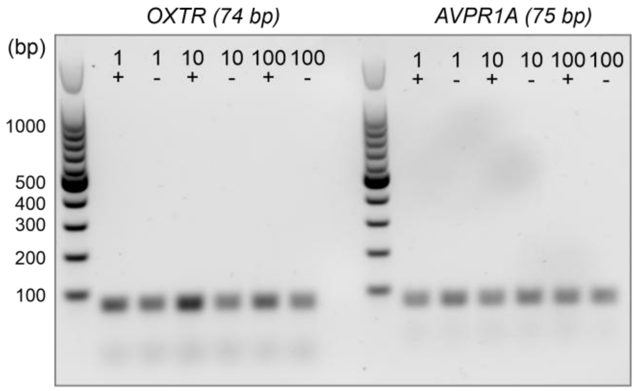

Figure S11. Full blot of RT-qPCR probe amplification products after FACS. Agarose (1.5%) resolved *OXTR* and *AVPR1A* qPCR products consisting of 74 and 75 base pairs (bp), respectively. Numbers at the top indicate nanomolar tracer used, with + and – denoting both positive and negative fluorescent cells sorted, respectively. n = 5,000 to 10,000 cells sorted per sample.

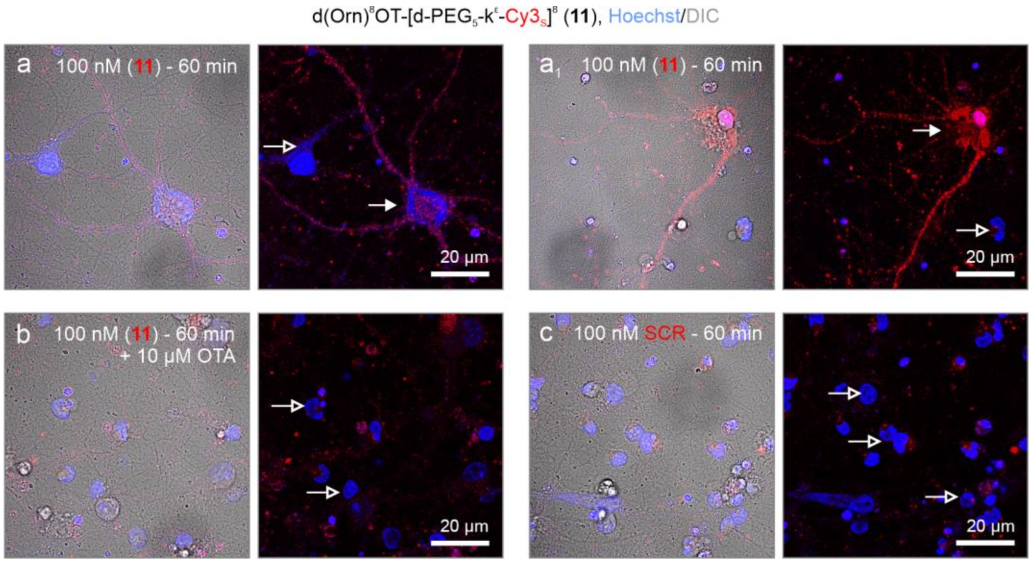

Figure S12. Compound uptake in primary rat hippocampal neurons. (**a,a$_1$**) Uptake of tracer (**11**) was found in a subpopulation of rat embryonic (E17.5) hippocampal neurons cultured for 14 days (*arrows vs. open arrows*). (**b,c**) Pretreatment with the peptide antagonist OTA prevented compound (**11**) internalization, while SCR was not taken up by neurons. Whole cells were visualized with differential interference contrast (DIC, in left figure panels) to reveal fine details and structures.

IV. Supporting Movies

Movie S1 and Movie S2. Live cell imaging showing the internalization of hOTR-GFP in HEK293 cells with either 10 nM (**Movie S1**, 17 min) or 100 nM (**Movie S2**, 14 min) tracer (**11**). Note the rapid internalization of both GFP and (**11**), as well as the strong cellular contraction, typical of $G_{q/11}$ engagement downstream of OTR.

Movie S3. **Raw Low-Resolution single particle tracking acquisition.** Representative low-resolution TIRFM of HEK293 or HEK293 expressing hOTR-GFP (HEK293-hOTR-GFP) cells imaged at 50 Hz following the addition of 1 nM of d(Orn)^{8}OT-[d-PEG$_5$-Cy5$_s$]^8 (**10**). Detections appear briefly and can be tracked to generate super-resolved images and quantify diffusion. 1000 frames of an acquisition are shown for each condition at 50 frames per second (fps). Scale bars 5 µm.

V. References

[1] T. Kremsmayr, M. Muttenthaler, "Fmoc Solid Phase Peptide Synthesis of Oxytocin and Analogues" *Methods Mol. Biol.* **2022**, *2384*, 175-199.

[2] O. Al Musaimi, A. Basso, B. G. de la Torre, F. Albericio, "Calculating Resin Functionalization in Solid-Phase Peptide Synthesis Using a Standardized Method based on Fmoc Determination" *ACS Comb. Sci.* **2019**, *21*, 717-721.

[3] S. Eissler, M. Kley, D. Bächle, G. Loidl, T. Meier, D. Samson, "Substitution determination of Fmoc-substituted resins at different wavelengths" *J. Pept. Sci.* **2017**, *23*, 757-762.

[4] M. Busnelli, G. Kleinau, M. Muttenthaler, S. Stoev, M. Manning, L. Bibic, L. A. Howell, P. J. McCormick, S. Di Lascio, D. Braida, M. Sala, G. E. Rovati, T. Bellini, B. Chini, "Design and Characterization of Superpotent Bivalent Ligands Targeting Oxytocin Receptor Dimers via a Channel-Like Structure" *J. Med. Chem.* **2016**, *59*, 7152-7166.

[5] T. Kremsmayr, A. Aljnabi, J. B. Blanco-Canosa, H. N. T. Tran, N. B. Emidio, M. Muttenthaler, "On the Utility of Chemical Strategies to Improve Peptide Gut Stability" *J. Med. Chem.* **2022**, *65*, 6191-6206.

[6] A. C. Conibear, N. L. Daly, D. J. Craik, "Quantification of small cyclic disulfide-rich peptides" *Biopolymers*. **2012**, *98*, 518-524.

[7] O. Trott, A. J. Olson, "AutoDock Vina: improving the speed and accuracy of docking with a new scoring function, efficient optimization, and multithreading" *J. Comput. Chem.* **2010**, *31*, 455-461.

[8] G. Wolber, T. Langer, "LigandScout: 3-D pharmacophores derived from protein-bound ligands and their use as virtual screening filters" *J. Chem. Inf. Model.* **2005**, *45*, 160-169.

[9] Y. Waltenspühl, J. Ehrenmann, S. Vacca, C. Thom, O. Medalia, A. Plückthun, "Structural basis for the activation and ligand recognition of the human oxytocin receptor" *Nat. Commun.* **2022**, *13*, 4153.

[10] Y. Cheng, W. H. Prusoff, "Relationship between the inhibition constant (K1) and the concentration of inhibitor which causes 50 per cent inhibition (I50) of an enzymatic reaction" *Biochem. Pharmacol.* **1973**, *22*, 3099-3108.

[11] M. Busnelli, E. Bulgheroni, M. Manning, G. Kleinau, B. Chini, "Selective and potent agonists and antagonists for investigating the role of mouse oxytocin receptors" *J. Pharmacol. Exp. Ther.* **2013**, *346*, 318-327.

[12] A. Oksche, G. Leder, S. Valet, M. Platzer, K. Hasse, S. Geist, G. Krause, A. Rosenthal, W. Rosenthal, "Variant amino acids in the extracellular loops of murine and human vasopressin V2 receptors account for differences in cell surface expression and ligand affinity" *Mol. Endocrinol.* **2002**, *16*, 799-813.

[13] A. Kechkar, D. Nair, M. Heilemann, D. Choquet, J. B. Sibarita, "Real-time analysis and visualization for single-molecule based super-resolution microscopy" *PLoS One*. **2013**, *8*, e62918.

[14] S. F. Longfield, M. Mollazade, T. P. Wallis, R. S. Gormal, M. Joensuu, J. R. Wark, A. J. van Waardenberg, C. Small, M. E. Graham, F. A. Meunier, R. Martínez-Mármol, "Tau forms synaptic nano-biomolecular condensates controlling the dynamic clustering of recycling synaptic vesicles" *Nat. Commun.* **2023**, *14*, 7277.

[15] G. Giannone, E. Hosy, F. Levet, A. Constals, K. Schulze, A. I. Sobolevsky, M. P. Rosconi, E. Gouaux, R. Tampé, D. Choquet, L. Cognet, "Dynamic superresolution imaging of endogenous proteins on living cells at ultra-high density" *Biophys. J.* **2010**, *99*, 1303-1310.

[16] M. Joensuu, P. Syed, S. H. Saber, V. Lanoue, T. P. Wallis, J. Rae, A. Blum, R. S. Gormal, C. Small, S. Sanders, A. Jiang, S. Mahrhold, N. Krez, M. A. Cousin, R. Cooper-White, J. J. Cooper-White, B. M. Collins, R. G. Parton, G. Balistreri, A. Rummel, F. A. Meunier, "Presynaptic targeting of botulinum neurotoxin type A requires a tripartite PSG-Syt1-SV2 plasma membrane nanocluster for synaptic vesicle entry" *EMBO J.* **2023**, *42*, e112095.

[17] R. S. Gormal, P. Padmanabhan, R. Kasula, A. T. Bademosi, S. Coakley, J. Giacomotto, A. Blum, M. Joensuu, T. P. Wallis, H. P. Lo, S. Budnar, J. Rae, C. Ferguson, M. Bastiani, W. G. Thomas, E. Pardon, J. Steyaert, A. S. Yap, G. J. Goodhill, M. A. Hilliard, R. G. Parton, F. A. Meunier, "Modular transient nanoclustering of activated β2-adrenergic receptors revealed by single-molecule tracking of conformation-specific nanobodies" *Proc. Natl. Acad. Sci. USA*. **2020**, *117*, 30476-30487.

[18] T. P. Wallis, A. Jiang, K. Young, H. Hou, K. Kudo, A. J. McCann, N. Durisic, M. Joensuu, D. Oelz, H. Nguyen, R. S. Gormal, F. A. Meunier, "Super-resolved trajectory-derived nanoclustering analysis using spatiotemporal indexing" *Nat. Commun.* **2023**, *14*, 3353.

[19] E. Muratspahić, N. Tomašević, S. Nasrollahi-Shirazi, J. Gattringer, F. S. Emser, M. Freissmuth, C. W. Gruber, "Plant-Derived Cyclotides Modulate κ-Opioid Receptor Signaling" *J. Nat. Prod.* **2021**, *84*, 2238-2248.
